# A Quality Control System for Automated Prostate Segmentation on T2-Weighted MRI

**DOI:** 10.3390/diagnostics10090714

**Published:** 2020-09-18

**Authors:** Mohammed R. S. Sunoqrot, Kirsten M. Selnæs, Elise Sandsmark, Gabriel A. Nketiah, Olmo Zavala-Romero, Radka Stoyanova, Tone F. Bathen, Mattijs Elschot

**Affiliations:** 1 Department of Circulation and Medical Imaging, NTNU—Norwegian University of Science and Technology, 7030 Trondheim, Norway; Kirsten.Margrete.Selnes@stolav.no (K.M.S.); gabriel.a.nketiah@ntnu.no (G.A.N.); tone.f.bathen@ntnu.no (T.F.B.); mattijs.elschot@ntnu.no (M.E.); 2 Department of Radiology and Nuclear Medicine, St. Olavs Hospital, Trondheim University Hospital, 7030 Trondheim, Norway; elise.sandsmark@stolav.no; 3 Department of Radiation Oncology, University of Miami Miller School of Medicine, Miami, FL 33136, USA; ozavala@coaps.fsu.edu (O.Z.-R.); rstoyanova@med.miami.edu (R.S.); 4 Center for Ocean-Atmospheric Prediction Studies, Florida State University, Tallahassee, FL 32306, USA

**Keywords:** prostate, segmentation, deep learning, radiomics, quality control, computer-aided detection and diagnosis, MRI, machine learning

## Abstract

Computer-aided detection and diagnosis (CAD) systems have the potential to improve robustness and efficiency compared to traditional radiological reading of magnetic resonance imaging (MRI). Fully automated segmentation of the prostate is a crucial step of CAD for prostate cancer, but visual inspection is still required to detect poorly segmented cases. The aim of this work was therefore to establish a fully automated quality control (QC) system for prostate segmentation based on T2-weighted MRI. Four different deep learning-based segmentation methods were used to segment the prostate for 585 patients. First order, shape and textural radiomics features were extracted from the segmented prostate masks. A reference quality score (QS) was calculated for each automated segmentation in comparison to a manual segmentation. A least absolute shrinkage and selection operator (LASSO) was trained and optimized on a randomly assigned training dataset (N = 1756, 439 cases from each segmentation method) to build a generalizable linear regression model based on the radiomics features that best estimated the reference QS. Subsequently, the model was used to estimate the QSs for an independent testing dataset (N = 584, 146 cases from each segmentation method). The mean ± standard deviation absolute error between the estimated and reference QSs was 5.47 ± 6.33 on a scale from 0 to 100. In addition, we found a strong correlation between the estimated and reference QSs (rho = 0.70). In conclusion, we developed an automated QC system that may be helpful for evaluating the quality of automated prostate segmentations.

## 1. Introduction

Prostate cancer is one of the most commonly diagnosed cancers among men worldwide [1]. Precise diagnosis is essential for management of the disease, where early detection and staging can increase the survival rate [2]. The current diagnostic process includes measuring elevated prostate-specific antigen (PSA) in the blood followed by prostate biopsy sampling and histopathology analysis. The addition of multiparametric magnetic resonance imaging (mpMRI) and the establishment of international guidelines for the image acquisition and interpretation have improved the diagnostic precision for prostate cancer [3,4]. However, the traditional, qualitative radiological interpretation of the images has a number of limitations, such as high inter-observer variability [5], its time-consuming nature and a lack of scalability of the manual data handling approach with increasing demand [6,7].

Automated computer-aided detection and diagnosis (CAD) systems, which exploit the quantitative information in MR images, are providing promising solutions to overcome these limitations of qualitative image interpretation and support clinical decision making [6,8]. Typically, the segmentation of the organ of interest, in this case the prostate gland, constitutes one of the first important steps in a CAD system workflow [7,9]. This step helps remove irrelevant image information and facilitates subsequent extraction of quantitative image features (radiomics) from sub-regions/volumes such as tumors for further analysis or diagnosis. However, manual segmentation of the prostate, which is traditionally performed on T2-weighted (T2W) MR images by radiologists, is a time-consuming task. Fortunately, recently developed segmentation algorithms have shown great promise to fully automate this step [10,11,12,13,14,15], which would save valuable time and could facilitate the integration of CAD systems in clinical practice.

Deep learning-based methods seem to be the most promising for this purpose, as they outperform the more traditional methods in the PROMISE12 prostate segmentation grand challenge [16] (https://promise12.grand-challenge.org/evaluation/results). Interestingly, the top-performing methods in this challenge scored better—on average—than a non-expert second reader. Nevertheless, none of the proposed methods is perfect. Occasionally, each of the proposed segmentation methods results in a few cases with unpredictable, suboptimal contours. Time-consuming manual verification of the contours by radiologists is thus still a necessary step, which limits the implementation of automated prostate segmentation algorithms in clinical practice. A quality control (QC) system that automatically provides an assessment of the segmentation quality could help overcome this limitation and standardize decisions about segmentation quality.

The aim of this study was to develop a fully automated QC system that generates a quality score for assessing the accuracy of automated prostate segmentations on T2W MR images. We trained, optimized and tested the proposed QC system using two data cohorts and four different deep learning-based segmentation algorithms. We explored the importance of the radiomics features the system is based on and compared a generalizable model with models trained on specific combinations of dataset and segmentation algorithm. Finally, we show that the quality of the segmentations can be successfully estimated by our QC system. 

## 2. Materials and Methods

We propose a novel QC system, which is designed to automatically score the quality of prostate segmentations on T2W MR images. Briefly, the inputs to the QC system are the T2W MR image and the corresponding deep learning-based prostate segmentation. Radiomics features are extracted from the segmented prostate image volume and fed into a least absolute shrinkage and selection operator (LASSO) to build a linear regression model [17], which is trained to generate an estimated quality score (eQS). Reference quality scores (rQSs) based on manual segmentations from experts are then used to assess the performance of the QC system.

### 2.1. Dataset

In this study, the PROMISE12 grand challenge [16] training dataset (N = 50) was only used to train and validate four different deep learning-based networks to segment three-dimensional (3D) prostate volumes on T2W MR images. This dataset consists of multi-center and multi-vendor transverse T2W MR images obtained with different acquisition protocols, field strengths and coils. Each of the trained networks was subsequently used to segment T2W MR images from the PROSTATEx challenges [18] (N = 346; seven cases excluded due to technical errors) and a dataset of in-house collected T2W MR images (N = 246), resulting in a combined dataset (N = 585). The combined dataset was shuffled and randomly split, in a controlled way, to ensure similar data distribution, into a training dataset (75%, N = 439) and a testing dataset (25%, N = 146) to respectively train/optimize and test the proposed QC system. 

The in-house collected dataset was obtained from St. Olavs Hospital, Trondheim University Hospital, Trondheim, Norway between March 2015 and December 2017. It consists of pre-biopsy 3T images from 246 patients (median age = 65; range: 44–76 years). T2W imaging was performed on a Magnetom Skyra 3T MRI system (Siemens Healthineers, Erlangen, Germany) with a turbo spin-echo sequence (repetition time/echo time = 4450–9520/101–108 ms, 320 × 320–384 × 384 matrix size, 26–36 slices, 3 mm slice thickness and 0.5 × 0.5–0.6 × 0.6 mm^2^ in plane resolution).

The Regional Committee for Medical and Health Research Ethics (REC Mid Norway) approved the use of the in-house collected dataset (identifier 2017/576; 5 May 2017) and granted permission for passive consent to be used. The two other datasets (PROMISE12 and PROSTATEx) were publicly available and details can be found in [16,18].

### 2.2. Prostate Segmentation

For each dataset, manual segmentations of the prostate gland without seminal vesicles were used as the gold standard. The PROMISE12 training dataset segmentations, used for training the segmentation algorithms, were publicly available [16]. The segmentation for the PROSTATEx dataset was performed by imaging experts with more than 25 years′ combined expertise in prostate imaging and reviewed by radiation oncologists at Miller School of Medicine, Miami, FL, USA. The in-house collected dataset segmentation was performed by a radiology resident (E.S.) at St. Olavs Hospital, Trondheim University Hospital, Trondheim, Norway, under the supervision of a radiologist with more than 10 years′ experience in prostate imaging. The manual segmentations of the PROSTATEx and in-house collected dataset were used to calculate the rQSs (see 2.3. Reference Quality Scores).

The deep learning-based prostate segmentation was performed using four different convolutional neural networks (CNNs), which are all variants of the famous U-Net with skip connections [15], here further referred to as U-Net [19], V-Net [10], nnU-Net-2D [11] and nnU-Net-3D [11]. U-Net and nnU-Net-2D perform the segmentation on a 2D slice-by-slice basis, whereas V-Net and nnU-Net-3D perform the segmentation on a 3D volume basis. Prior to segmentation, all images were pre-processed in accordance with the corresponding segmentation method. The segmentation pre-processing and the network training, validation and testing were performed on a single NVIDIA Tesla P100 PCIe 16 GB GPU in Ubuntu 16.04.6 LTS system. U-Net was implemented with the Keras API (version 2.3.0; https://keras.io) backboned with TensorFlow (version 1.9.0; https://www.tensorflow.org) using Python (version 2.7.12; Python Software Foundation, Wilmington, DE, USA). V-Net, nnU-Net-2D and nnU-Net-3D were implemented with PyTorch (version 1.4.0; https://pytorch.org) using Python (version 3.6.9).

### 2.3. Reference Quality Scores

To assess the true quality of the automated segmentations, rQSs were calculated in accordance with Litjens et al. [16]. Briefly, the rQS is a combination of the dice similarity coefficient (DSC) [20], the absolute relative volume difference (aRVD) [21], the 95% Hausdorff distance (95HD) [22] and the average symmetric surface distance (ASD) [21], separately obtained from the whole prostate, apex and base by comparing the automated segmentations with the manual segmentations (gold standard). Here, we defined the apex and base of the prostate to be the inferior and superior third parts of the mask-containing slices, respectively. However, before these 12 metrics can be combined in a single rQS, they need to be transformed to a common scale [16,21]. To do this, a second observer (M.R.S.S., three years of experience with prostate imaging) manually segmented 50 randomly selected cases from the combined dataset. These cases were used to calculate, for each metric, a linear function that maps the metric on a scale from 0 to 100, with the average performance of the second observer fixed at 85. These linear functions were subsequently applied to the 12 metrics calculated for each automated segmentation and the resulting 12 scores were averaged to obtain a single rQS for each segmentation. Details are provided in Appendix A.

### 2.4. Quality Control System

**Figure 1:**
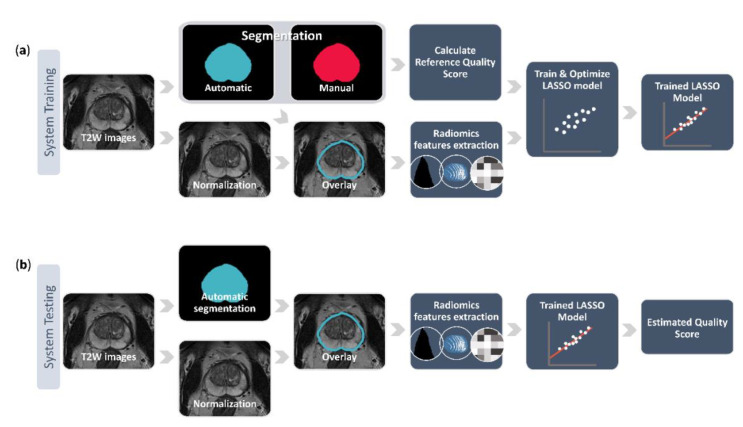
The pipeline of training (**a**) and testing (**b**) the proposed quality control system. The system training starts from the normalized T2-weighted (T2W) image stack with its corresponding manual prostate segmentation and automated segmentation delivered by a deep learning-based segmentation method. These two segmentations are used to calculate the reference quality score and the automated segmentation is also overlaid on the normalized image stack to extract various radiomics features. The reference quality score and the features are then fed into a least absolute shrinkage and selection operator (LASSO) to train and optimize a linear regression model that predicts the quality scores based on the imaging features. During the system testing, the trained model uses the radiomics features extracted from the overlaid automated segmentation on the normalized image stack to estimate a quality score for a previously unseen case.

**Figure 2:**
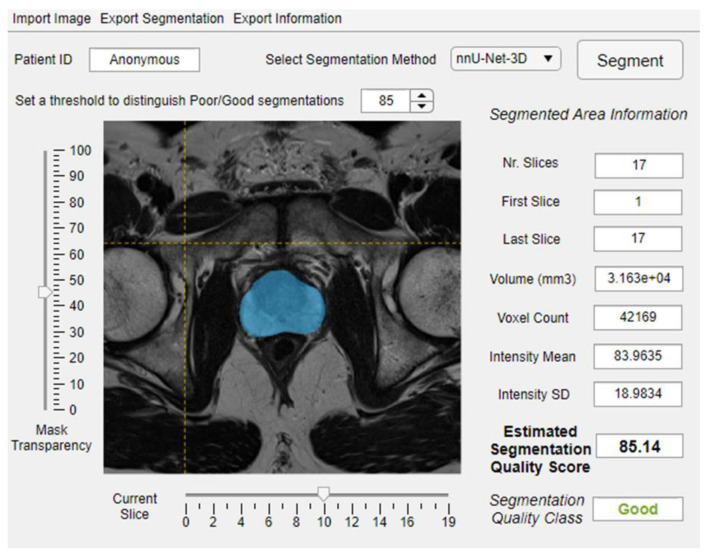
An example of integrating the proposed quality control system within image analysis software.

Figure 1 gives an overview of the proposed QC system. After preprocessing the T2W images, the LASSO model was trained and optimized on the training dataset, and tested on the independent testing dataset. All steps were implemented in MATLAB R2019b (MathWorks, Natick, MA, USA), except for the feature extraction which was performed using Python (version 3.7.3). The proposed system will be made available on GitHub at https://github.com/ntnu-mr-cancer/SegmentationQualityControl. Figure 2 shows an example of how the proposed QC system can be integrated in the image analysis pipeline. 

#### 2.4.1. Data Preparation

All T2W images were N4 bias field corrected [23] and intensity normalized using the AutoRef method [24]. In an attempt to develop a generalizable QC model, the segmentations generated by the four CNNs were combined in one dataset, producing a system training dataset of n = 1756 images (439 images from each CNN) and a system testing dataset of n = 584 images (146 images from each CNN) with corresponding segmentations. The dataset was split on the patient level, so all four segmentations belonging to one patient ended up in either the system training dataset or the system testing dataset.

Feature extraction from the preprocessed T2W images was performed using the automated prostate segmentations as the region of interest. All the features were extracted using Pyradiomics (version 2.2.0; an open-source Python package) [25]. Discretization of image intensity was performed using the fixed bin size approach, as recommended by Pyradiomics. The bin width was set to 64 in correspondence with the relatively large volume of interest. The features (N = 107) consisted of first-order features (N = 18), shape features (N = 14, performed on prostate 3D volume) and texture features (N = 75, 24 features from the gray level co-occurrence matrix (GLCM; in 3D along 13 directions (26-connectivity) and 1 pixel distance) [26], 16 features from the gray level run length matrix (GLRLM; in 3D along 13 directions) [27], 16 features from the gray level size zone matrix (GLSZM; in 3D along 13 directions) [28], 14 features from the gray level dependence matrix (GLDM; 1 pixel distance) [29] and 5 features from the neighboring gray tone difference matrix (NGTDM; 1 pixel distance) [30]). The average of the GLCM, GLRLM and GLSZM features across the direction was used. A complete list of the extracted features is given in Table S1. The features were extracted from the 3D volume of the whole prostate, apex and base parts of the prostate, separately, giving a total of 321 features per case.

#### 2.4.2. Model Training, Optimizing and Testing

A least absolute shrinkage and selection operator (LASSO) [17] was used to build a linear regression model. The model was trained using the extracted features (N = 321) as predictors and the rQSs as responses. The LASSO, by nature, performs feature selection to enhance the model accuracy and interpretability [31]. How many features are selected depends on the regularization parameter lambda, which needs to be optimized. We employed a 5-fold cross-validation scheme to find the optimal lambda, here defined as the model returning the lowest mean squared errors between the eQS and rQS while satisfying a non-biased distribution as visualized by Bland–Altman plots [32].

The optimized model was tested and evaluated on the system testing dataset, returning an eQS for each segmentation based on features extracted from the deep learning-based prostate segmentation in the T2W MR image. If the returned eQS was > 100 it was set to 100 and if it was < 0 it was set to 0. The mean absolute error (MAE) and Spearman’s rank test between eQSs and rQSs were used to evaluate the performance of the QC system. This was done on all the cases of the system testing dataset (General model), as well as separately for each of the eight combinations of dataset and segmentation method (sub-results from the General model; e.g., PROSTATEx—U-Net). The sub-results from the General model were also compared to the performance of (non-generalizable) models specifically trained on each combination of dataset and segmentation method. The manual and automated segmentations belonging to outliers of the tested General model were visually inspected by a researcher with three years of experience with prostate imaging (M.R.S.S.).

## 3. Results

### 3.1. Reference Quality Scores

**Figure 3:**
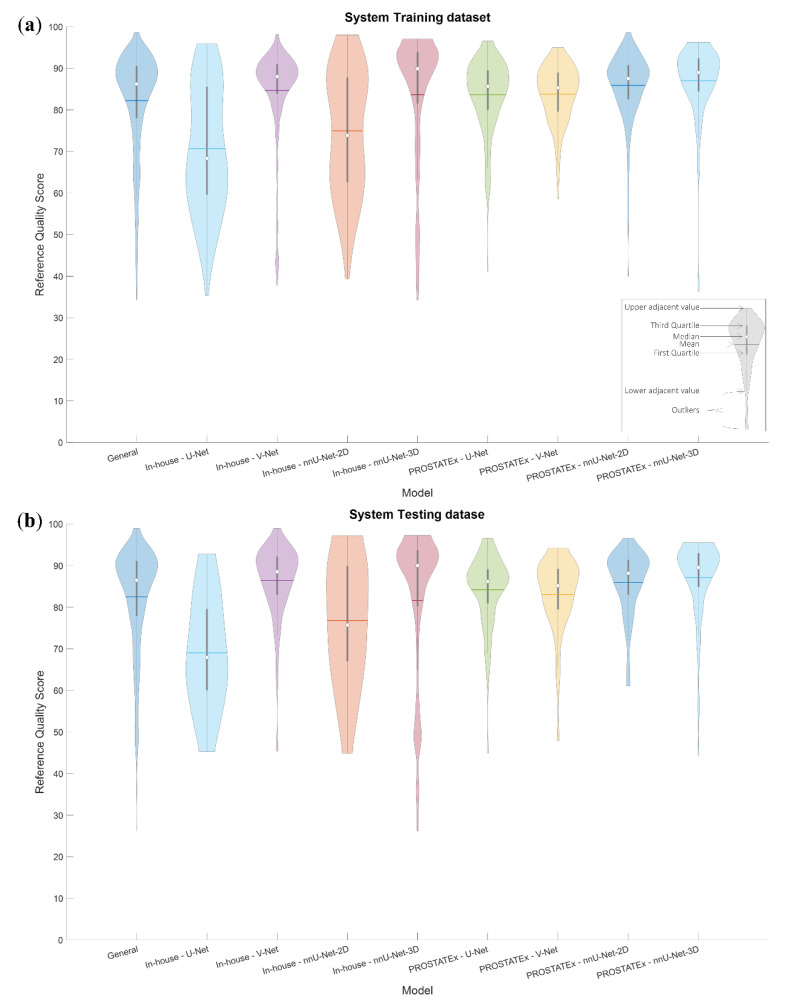
Violin plots visualizing the distribution of the reference quality scores for the system training (**a**) and testing (**b**) datasets, both combined and for each combination of dataset and segmentation method.

The rQSs of the system training and testing dataset segmentations are presented in Figure 3. The maximum, mean ± standard deviation and minimum rQS of the combined dataset were 98.65, 82.26 ± 12.19 and 34.24, respectively, for the system training dataset and 98.95, 82.51 ± 12.22 and 26.24, respectively, for the system testing dataset. Figure 3 shows that the distribution of rQSs varies both between datasets and among the segmentation methods, indicating that the performance of automated prostate segmentation depends on both the dataset and the method used.

### 3.2. Training and Optimization

The maximum, mean ± standard deviation and minimum eQS of the General model were 98.04, 82.26 ± 10.71 and 28.06, respectively, for the system training dataset.

**Figure 4:**
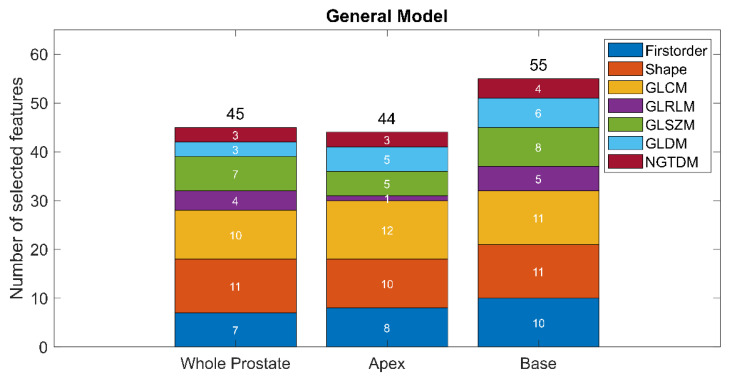
The distribution of the selected features in the optimized General model.

The optimal lambda was found to be 0.01, which resulted in the selection of 142 out of 321 radiomics features in the trained General LASSO model. Figure 4 shows the distribution of the selected features. Overall, 46.30%, 76.19%, 45.83%, 20.83%, 41.67%, 33.34% and 66.67% of the extracted first order, shape, GLCM, GLRLM, GLSZM, GLDM and NGTDM features were selected, respectively. Further details of the trained model are provided in Table S2. The details of the eight non-generalizable models are provided in Tables S3–S10.

**Figure 5:**
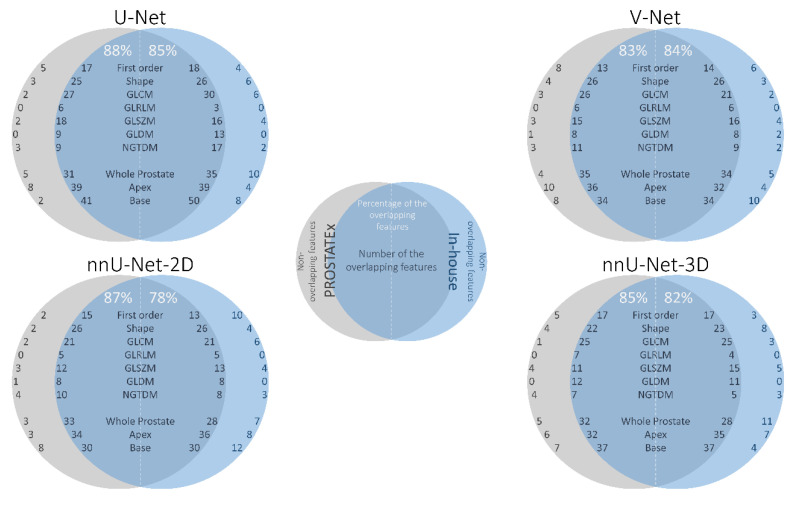
The overlap between the features in the PROSTATEx (gray) and in-house (blue) datasets of the same segmentation method (e.g., overlap between the “PROSTATEx—U-Net model” and “In-house—U-Net model”). The intersection area presents the overlapping features, whereas the areas out of the intersection present the set of features unique to each dataset.

Figure 5 shows the overlap between the selected features in the PROSTATEx and in-house datasets of non-generalizable models trained on data processed with the same segmentation method. To account for the high co-linearity between features, overlap was defined as the selection of the same feature or a highly correlated feature (rho > 0.9). For each segmentation method, we found a high number of overlapping features (directly or highly correlated), indicating that the models extracted similar features irrespective of dataset. 

### 3.3. Testing

For the system testing dataset, the maximum, mean ± standard deviation and minimum eQS of the General model were 97.60, 82.03 ± 11.02 and 0.00, respectively.

The performance of the tested models is presented in Table 1. Table 2 presents sub-results from the tested General model, for direct comparison with the non-generalizable models. Sub-results from the General model resulted in lower MAE in 7/8 cases than their non-generalizable counterparts, indicating that the overall performance of the General model is better than the non-generalizable models. Nevertheless, it should be noted that the sub-results vary considerably. This is especially apparent from the difference in slope (ideally 1), intercept (ideally 0) and rho (ideally 1) between results from the PROSTATEx and in-house datasets.

**Figure 6:**
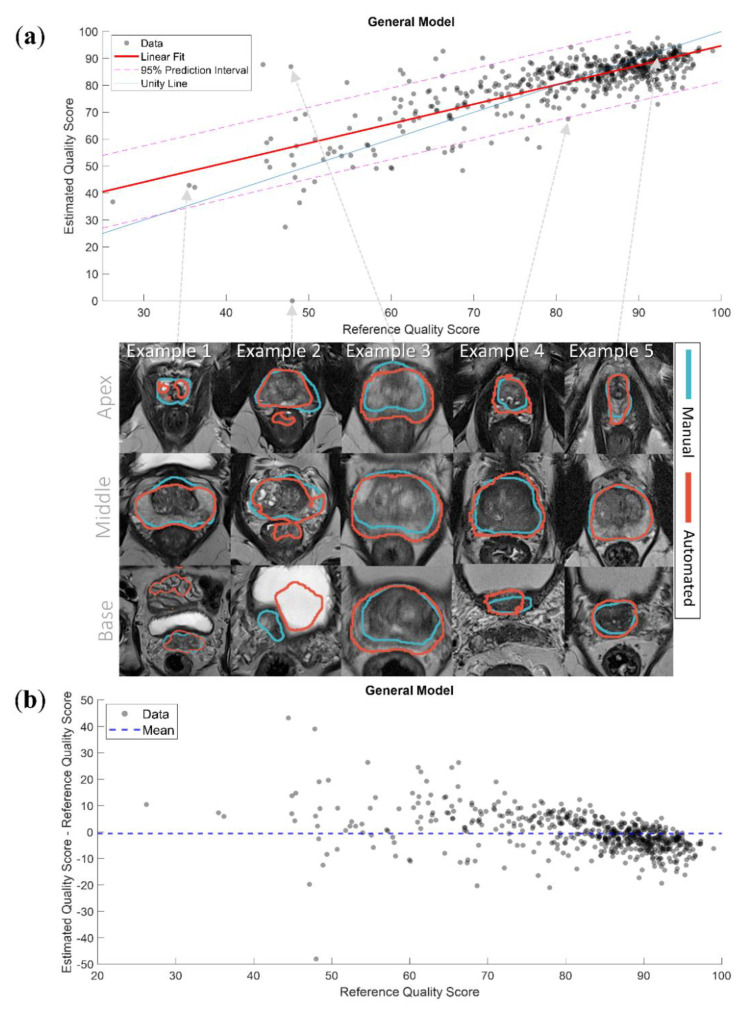
(**a**) The linear fit of the estimated quality scores with 95% prediction interval of the General model with examples of segmentations. Example 1 presents a case where the model accurately predicted the quality score (QS) of a low-quality automated segmentation; Example 2 presents a case where the model extremely underestimated the QS of a low-quality automated segmentation, the automated segmentation here covered parts of the rectum and the bladder; Example 3 presents a case where the model extremely overestimated the QS of a low-quality automated segmentation, the manual segmentation here misses the peripheral zone; Example 4 presents a case where the model slightly underestimated the QS of a high-quality automated segmentation, the automated segmentation here was slightly over segmented; Example 5 presents a case where the model accurately predicted the QS of a high-quality automated segmentation; (**b**) the difference between the estimated and reference quality scores of the General model.

Figure 6a shows the linear fit of the eQSs for the General model with examples of segmentations. The segmentations of the cases outside of the 95% prediction interval were visually inspected. We subjectively judged the eQS to be extremely overestimated in 2/9 segmentations that were over the 95% prediction interval, and extremely underestimated in 3/18 segmentations that were under the 95% prediction interval. The rest of the visually inspected segmentations were judged to have an eQS that acceptably represented the quality of the automated segmentation. All of the segmentations over the 95% prediction interval belonged to the PROSTATEx dataset, and all of the segmentations under the 95% prediction interval belonged to the in-house dataset. Interestingly, in 8/27 segmentations, the discrepancy between the eQS and rQS was judged to result from a sub-optimal manual segmentation. Examples of over- and underestimated segmentations are shown in Figure 6a. Figure 6b shows the difference between the eQSs and rQSs of the General model. The mean difference was −0.48, with a tendency for overestimating cases with a low rQS and underestimating cases with a high rQS.

**Figure 7:**
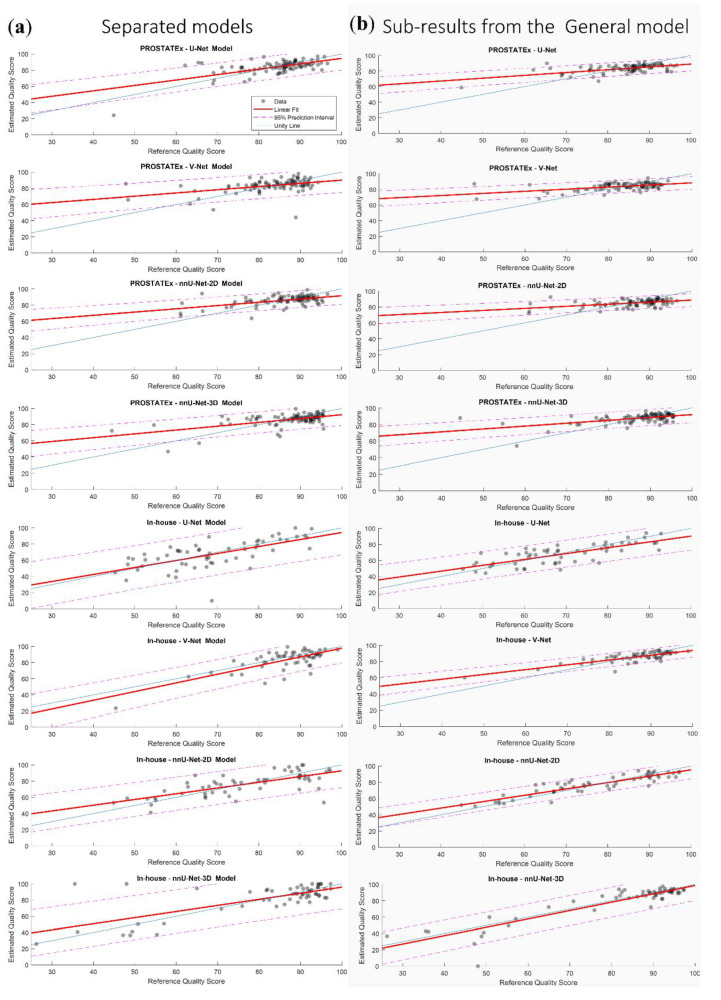
The linear fit of the estimated quality scores with 95% prediction interval of the eight non-generalizable models (**a**) and the sub-results from the General model (**b**).

The linear fits of the eight non-generalizable models and the sub-results from the General model are presented in Figure 7. It can be appreciated that the slopes and intercepts of the models/sub-results associated with the in-house dataset were better than those associated with the PROSTATEx dataset.

## 4. Discussion

Automated segmentation of the prostate is a crucial step in the CAD of prostate cancer, but quality control and possibly adjustment by a trained radiologist is still required. In this work, we present a fully automated QC system that aims to present the user with an estimated score indicative of the segmentation quality. This system could function as a safety net that saves time and costs, standardizes the decision about the segmentation accuracy and thus facilitate the clinical implementation of automated prostate segmentation algorithms. The system could be specifically useful for clinical applications that are sensitive to errors in segmentation, such as MRI–ultrasound fusion for targeted prostate biopsies, which is currently becoming a clinical standard procedure [33], and prostate-targeted MR-guided radiotherapy, which has been implemented in the treatment of prostate cancer patients during the last few years [34].

Our results indicate that the proposed QC system could be helpful for this purpose. Overall, the General model had better performance than the non-generalizable models. We found a strong correlation between the rQSs and eQSs (rho = 0.70) and MAE values less than the standard deviation between the experts and the second observer segmentations (5.37 vs. 7.76), implying that errors were in an acceptable range. In addition, the mean of the differences between the eQSs and rQSs was low (mean = −0.48). Despite the overall good performance, some of the eQSs of the segmentations were over- or underestimated. This can be partly explained by the fact that the rQSs, used as input for training the model, were imbalanced and skewed towards high scores. This probably had an effect on the model performance, leading to a higher number of over- and underestimated segmentations around the low rQSs. Indeed, the non-generalizable models that had the most balanced distribution of rQSs in the training dataset (e.g., “In-house—U-Net” and “In-house—nnU-Net_2D”) performed better than the other models.

T2W MRI clearly depicts the borders and anatomy of the prostate gland, and thus constitutes an excellent starting point for both prostate segmentation algorithms and the proposed QC system. In this work, we implemented four deep learning-based segmentation methods using two different T2W MRI datasets. Combining these datasets made the proposed system more generalizable and robust, and it is thus potentially applicable to other segmentation methods and datasets. However, we also showed that the model did not perform well for all combinations of dataset and segmentation method. Consequently, the proposed QC system should be carefully tested and evaluated on new data and methods before application.

The first-order, shape and texture features were investigated because they describe distinct characteristics of the volume of interest. Our QC system was trained to find common features and assess the segmentation quality among the investigated cases. We selected the LASSO model due to its model interpretability advantage [31] and its good performance in multiple radiomics studies [35,36,37]. To calculate the rQS, we chose to use the established PROMISE12 challenge evaluation metric [16] as it imparts a comprehensive overview of the segmentation accuracy, and shows interest in the prostate apex and base segmentations, which are the most difficult parts of the prostate gland to segment. It is paramount to segment these two sections correctly in some of the clinical applications and procedures, e.g., in MRI–ultrasound fusion for targeted prostate biopsy [33]. Similar to Litjens et al. [16], the average performance of the second observer was fixed at 85 during the rQS calculation due to the relatively good correspondence between the second observer segmentations and the gold standard.

To develop a flexible system, we chose to train a regression model instead of a classifier. A classifier would require a fixed threshold to distinguish the good and poor rQSs, which is challenging and depends on the targeted clinical application. Moreover, a fixed threshold may restrict the system’s generalizability. Depending on the desired application and corresponding acceptable segmentation error margin, a threshold can be set to distinguish poor from acceptable segmentations (e.g., Figure 2). The QC system could thus save time for radiologists, as many of the segmentations can be used without further manual verification; the total computational time to generate an automated segmentation and corresponding eQS was less than one minute per case on the described computing system, which is drastically less than the time required by a radiologist to do the same task. In addition, the system could standardize the decision about the segmentation accuracy and build confidence using the deep learning-based algorithms. 

All the different types of features available in Pyradiomics were used in the trained General model. However, shape features were found to be the most important, since approximately 76% of them were selected. This finding is in accordance with the way the CNNs work, gradually moving from shape-based to texture-based features through the layers [38]. Interestingly, compared to features extracted from the apex and the whole prostate, a higher number of features was selected from the base of the prostate for the model training. This potentially reflects how difficult it is to segment—both manually and automatically—the prostate base due to the variability between patients [16].

It could be noticed from Figure 3 that the rQS distributions are wider and more balanced in the case of the in-house dataset. This has a positive effect on the performance of the non-generalizable models as well as the General model’s sub-results associated with the in-house dataset. This is especially noticeable from the low eQSs, which are closer to the unity line than those associated with the PROSTATEx dataset. The high overlap between the features of the non-generalizable models trained on the PROSTATEx and the in-house datasets indicates that the performance difference is probably due to the input data, and not caused by differences in the selected dataset features. For future work, the model performance could potentially be enhanced by increasing the number of low rQSs by stopping the CNN training early, i.e., before finishing the recommended number of iterations.

Despite the acceptable performance of the QC system General model, there were some outliers, here defined as the eQSs outside the 95% prediction interval limits. Visual inspection revealed that the eQSs of the segmentations actually accurately represented the quality of the automated segmentations for most of these over- and underestimated cases. It was found that 8/9 segmentations over the 95% prediction interval outliers belonged to the four CNN segmentations of two patients. The manual segmentation of one of these patients was missing the contour in some of the slices in the apex and the base, and not properly covering the peripheral zone in the middle part of the prostate gland (see Example 3 in Figure 6a). The manual segmentation of the other patient did not include the peripheral zone in all slices from base to middle prostate. The automated segmentations associated with an underestimated eQS included in many cases small areas outside of the regions of interest (see Example 2 in Figure 6a). The visual inspection also revealed that all of the overestimated cases belonged to the PROSTATEx dataset and all of the underestimated cases belonged to the in-house dataset, which might be explained by the distribution of the rQSs used in training the system.

In this study, we propose a QC system that estimates the quality of automated prostate segmentations based on the shape of the segmentation mask, and the histogram intensity and texture of the underlying T2W image. Another interesting approach, which requires an additional step, was recently proposed by Valindria et al. [39]. In their reverse classification accuracy method, a segmentation model was built from the segmentation mask and corresponding image of a single new case (lacking ground truth). Subsequently, this model was applied to all images of a database with corresponding expert segmentations. Under the assumption that the same segmentation model should work for at least one of these images, the best segmentation accuracy (DSC) is assumed to reflect the accuracy of the newly segmented case. Robinson et al. [40] showed that this approach works well for QC of segmentation of the heart in cardiovascular MRI. Yet another interesting approach was recently presented by Roy et al. [41], in which a structure-wise uncertainty estimate was intrinsically included in a CNN algorithm for brain segmentation on T1 MR images. This approach keeps the drop-out layers of the CNN active during test time, to produce multiple segmentation variants from which uncertainty measures can be calculated. One disadvantage compared to our system is that Roy et al.’s approach cannot be easily generalized to other segmentation methods. Moreover, unlike our system, both of the aforementioned approaches used only volume-based segmentation accuracy metrics and did not take boundary-based metrics into consideration. To the best of our knowledge, these methods have not yet been tested for prostate segmentation. Although they are more complex, it will be interesting to compare them with our system in future work.

Our study has limitations. The number of cases with a low rQS was relatively small; a more balanced dataset would probably have led to a more robust system over all datasets and segmentation methods and would have given better insight into the system’s potential. In addition, there are other radiomics features such as wavelet transformation-based texture features, which were not include in our model. These features could potentially enhance the performance of the system at the cost of generating a more complex model and expanding the computational time. For these reasons, they have not been used in this study, but their additional value will be investigated in future work.

## 5. Conclusions

We propose a QC system for estimating the quality of automated segmentation of the prostate in T2W MR images, which could be an important step towards the clinical implementation of computer-aided detection and diagnosis of prostate cancer. The performance of the generalizable model is acceptable in regard to estimating the segmentation quality scores, but varies between datasets and segmentation methods. The system is transparent and could save considerable time and standardize decision-making in clinical practice, albeit careful implementation and testing is required.

## Figures and Tables

**Table 1:** The performance evaluation of the separately tested models.

Model	N	MAE ± SD	IQR	Slope	Intercept	Rho	Correlation p-Value
General	584	5.37 ± 11.02	9.32	0.72	22.40	0.70	<0.001
PROSTATEx—U-Net	89	5.48 ± 9.04	7.20	0.67	27.83	0.49	<0.001
PROSTATEx—V-Net	89	5.91 ± 8.21	6.80	0.40	50.43	0.43	<0.001
PROSTATEx—nnU-Net-2D	89	5.14 ± 6.04	5.96	0.40	51.25	0.41	<0.001
PROSTATEx—nnU-Net-3D	89	5.89 ± 7.79	5.64	0.47	44.97	0.40	<0.001
In-house—U-Net	57	9.55 ± 17.24	22.95	0.86	7.92	0.70	<0.001
In-house—V-Net	57	6.58 ± 13.01	12.33	1.07	−9.55	0.55	<0.001
In-house—nnU-Net-2D	57	8.18 ± 14.2	21.26	0.71	21.99	0.67	<0.001
In-house—nnU-Net-3D	57	8.35 ± 19.02	14.78	0.75	20.73	0.48	<0.001

N: Number of segmentations; MAE: Mean absolute error; SD: Standard deviation of the absolute error; IQR: Interquartile range.

**Table 2:** Sub-results from the tested General model performance evaluation.

Sub-Results Combination	N	MAE ± SD	IQR	Slope	Intercept	Rho	Correlation *p*-Value
PROSTATEx—U-Net	89	5.24 ± 5.28	6.20	0.36	52.69	0.50	<0.001
PROSTATEx—V-Net	89	5.50 ± 4.67	5.33	0.27	61.28	0.38	<0.001
PROSTATEx—nnU-Net-2D	89	5.41 ± 4.46	5.37	0.26	62.80	0.43	<0.001
PROSTATEx—nnU-Net-3D	89	4.85 ± 5.76	6.12	0.35	57.17	0.50	<0.001
In-house—U-Net	57	7.27 ± 12.61	19.84	0.73	17.59	0.76	<0.001
In-house—V-Net	57	4.39 ± 6.64	6.47	0.59	34.65	0.70	<0.001
In-house—nnU-Net-2D	57	4.84 ± 12.4	17.78	0.78	16.90	0.87	<0.001
In-house—nnU-Net-3D	57	5.76 ± 20.79	10.17	1.02	−3.50	0.74	<0.001

N: Number of segmentations; MAE: Mean absolute error; SD: Standard deviation of the absolute error; IQR: Interquartile range.

## Supplementary Materials

The following are available online at https://www.mdpi.com/2075-4418/10/9/714/s1, Table S1: List of the extracted radiomics features. The shape features were extracted from the three-dimensional volume, Table S2: The trained General model intercept and coefficients, Table S3–S10: The trained non-generalizable models′ intercepts and coefficients.

## Appendix A

The metrics we used to calculate the rQSs, as mentioned in Section 2.3. Reference Quality Scores, are defined in Equations (A1), (A2), (A4) and (A5). Equation (A3) was required to enable the calculation of Equations (A4) and (A5). The linear mapping function that we used is defined in Equation (A6). All calculations are in accordance with Litjens et al. [16].

DSC (X,Y) = 2|X∩Y|/(|X| + |Y|) (A1)

where X and Y represent the reference and automated segmentation voxels, respectively.

aRVD (X,Y) = |(Y/X − 1) × 100| (A2)

to calculate 95HD and ASD, the Euclidean distance of the surface point sets (d_H_) from the reference (Xs) and automated (Ys) segmentations was measured using Equation (A3):

(A3) $$d_{H}\;(\mathrm{Xs},\mathrm{Ys})=\max_{x\in X_{s}}\left(\min_{y\in Y_{s}}\;d\left(x,y\right)\right)$$

where d is the Euclidean distance operator.

95HD = max (P_95_ (d_H_ (Xs,Ys)), P_95_ (d_H_ (Ys,Xs))) (A4)

where P_95_ represents the 95th percentile of d_H_.

(A5) $$\mathrm{ASD}\;(\mathrm{Xs},\mathrm{Ys})\;=\;(\sum_{x\in X_{s}}\min_{y\in Y_{s}}\;d\left(x,y\right)+\;\sum_{y\in Y_{s}}\min_{x\in X_{s}}\;d\left(y,x\right))/(N_{\mathrm{Xs}}+N_{\mathrm{Ys}})$$

where N_Xs_ and N_Ys_ represent the number of surface points of the reference and automated segmentations, respectively.

metric score (Z) = max (aZ + b, 0) (A6)

where Z is the average unmapped metric value. The variables a and b were determined by solving two equations through setting the metric score to equal 85, representing the average performance of the second observer, and a perfect score to equal 100.
