# Supplementary material for: A Quality Control System for Automated Prostate Segmentation on T2-Weighted MRI
Source: Diagnostics (Basel). 2020 Sep 18;10(9):714. doi: 10.3390/diagnostics10090714 (PMC7555425; doi:10.3390/diagnostics10090714)
Supplement: Supplementary file 1 [file diagnostics-10-00714-s001.pdf]

Table S1. List of the extracted Radiomics features. The shape features were extracted from the 3-dimensional volume.

First order	Shape
10Percentile	Elongation
90Percentile	Flatness
Energy	Least Axis Length
Entropy	Major Axis Length
Interquartile Range	Maximum 2D Diameter Column
Kurtosis	Maximum 2D DiameterRow
Maximum	Maximum 2D DiameterSlice
Mean Absolute Deviation	Maximum 3D Diameter
Mean	Mesh Volume
Median	Minor Axis Length
Minimum	Sphericity
Range	Surface Area
Robust Mean Absolute Deviation	Surface Volume Ratio
Root Mean Squared	Voxel Volume
Skewness	
Total Energy	
Uniformity	
Variance	

GLCM	GLRLM
Autocorrelation	Gray Level Non Uniformity
Cluster Prominence	Gray Level Non Uniformity Normalized
Cluster Shade	Gray Level Variance
Cluster Tendency	High Gray Level Run Emphasis
Contrast	Long Run Emphasis
Correlation	Long Run High Gray Level Emphasis
Difference Average	Long Run Low Gray Level Emphasis
Difference Entropy	Low Gray Level Run Emphasis
Difference Variance	Run Entropy
Inverse Difference	Run Length Non Uniformity
Inverse Difference Moment	Run Length Non Uniformity Normalized
Inverse Difference Moment Normalized	Run Percentage
Inverse Difference Normalized	Run Variance
Informational Measure of Correlation1	Short Run Emphasis
Informational Measure of Correlation2	Short Run High Gray Level Emphasis
Inverse Variance	Short Run Low Gray Level Emphasis
Joint Average	
Joint Energy	
Joint Entropy	
Maximal Correlation Coefficient	
Maximum Probability	
Sum Average	
Sum Entropy	
Sum Squares	

GLSZM	GLDM
Gray Level Non Uniformity	Dependence Entropy
Gray Level Non Uniformity Normalized	Dependence Non Uniformity
Gray Level Variance	Dependence Non Uniformity Normalized
High Gray Level Zone Emphasis	Dependence Variance
Large Area Emphasis	Gray Level Non Uniformity
Large Area High Gray Level Emphasis	Gray Level Variance
Large Area Low Gray Level Emphasis	High Gray Level Emphasis
Low Gray Level Zone Emphasis	Large Dependence Emphasis
Size Zone Non Uniformity	Large Dependence High Gray Level Emphasis
Size Zone Non Uniformity Normalized	Large Dependence Low Gray Level Emphasis
Small Area Emphasis	Low Gray Level Emphasis
Small Area High Gray Level Emphasis	Small Dependence Emphasis
Small Area Low Gray Level Emphasis	Small Dependence High Gray Level Emphasis
Zone Entropy	Small Dependence Low Gray Level Emphasis
Zone Percentage	
Zone Variance	

NGTDM	
Busyness	
Coarseness	
Complexity	
Contrast	
Strength	

Table S2. The trained General model intercept and coefficients.

Feature	Coefficient
Intercept	-589.649
firstorder_10Percentile_WP	-0.146612531
firstorder_90Percentile_WP	0.314
firstorder_Kurtosis_WP	-0.117812674
firstorder_Maximum_WP	-0.084492677
firstorder_Skewness_WP	0.593
firstorder_TotalEnergy_WP	1.02358E-08
firstorder_Uniformity_WP	230.905
shape_Elongation_WP	5.983
shape_Flatness_WP	4.539
shape_MajorAxisLength_WP	-0.06845567
shape_Maximum2DDiameterColumn_WP	-0.230560053
shape_Maximum2DDiameterRow_WP	-0.197985328
shape_Maximum2DDiameterSlice_WP	0.097
shape_Maximum3DDiameter_WP	-0.017992308
shape_Sphericity_WP	60.282
shape_SurfaceArea_WP	0.000246851
shape_SurfaceVolumeRatio_WP	14.199
shape_VoxelVolume_WP	1.89481E-05
glcm_Autocorrelation_WP	0.012
glcm_ClusterProminence_WP	2.54466E-06
glcm_ClusterShade_WP	-0.000307025

glcm_Correlation_WP	-6.007937184
glcm_Idmn_WP	399.583
glcm_Idn_WP	39.058
glcm_Imc2_WP	-2.099325012
glcm_MCC_WP	1.038
glcm_MaximumProbability_WP	399.127
glcm_SumSquares_WP	-0.009626445
glrlm_GrayLevelNonUniformity_WP	8.3781E-05
glrlm_RunEntropy_WP	-3.564375279
glrlm_ShortRunEmphasis_WP	169.42
glrlm_ShortRunLowGrayLevelEmphasis_WP	-94.32961035
glszm_GrayLevelNonUniformityNormalized_WP	-6.943523116
glszm_HighGrayLevelZoneEmphasis_WP	-0.000191147
glszm_LargeAreaEmphasis_WP	-7.997E-05
glszm_LargeAreaHighGrayLevelEmphasis_WP	-1.77614E-07
glszm_LowGrayLevelZoneEmphasis_WP	-826.0651781
glszm_SizeZoneNonUniformity_WP	0.00055125
glszm_SmallAreaEmphasis_WP	13.466
ngtdm_Coarseness_WP	-1711.077441
ngtdm_Complexity_WP	-1.5201E-05
ngtdm_Contrast_WP	-41.04467909
gldm_LargeDependenceHighGrayLevelEmphasis_WP	-0.001140483
gldm_LargeDependenceLowGrayLevelEmphasis_WP	2.304
gldm_SmallDependenceLowGrayLevelEmphasis_WP	4037.305
firstorder_10Percentile_Apex	0.07
firstorder_90Percentile_Apex	0.005208716
firstorder_InterquartileRange_Apex	-0.167191767
firstorder_Kurtosis_Apex	-1.413813301
firstorder_Maximum_Apex	0.002036756
firstorder_Range_Apex	0.022
firstorder_TotalEnergy_Apex	3.86153E-09
firstorder_Variance_Apex	-0.003263068
shape_Flatness_Apex	4.344
shape_LeastAxisLength_Apex	-0.25878969
shape_MajorAxisLength_Apex	-0.181309904
shape_Maximum2DDiameterColumn_Apex	0.144
shape_Maximum2DDiameterRow_Apex	-0.05676138
shape_Maximum2DDiameterSlice_Apex	-0.160179193
shape_Maximum3DDiameter_Apex	0.128
shape_MinorAxisLength_Apex	-0.078853106
shape_Sphericity_Apex	-18.44345633
shape_SurfaceVolumeRatio_Apex	-8.651565013
glcm_Autocorrelation_Apex	0.002396675
glcm_ClusterProminence_Apex	1.58546E-05
glcm_ClusterShade_Apex	-0.000233358
glcm_Contrast_Apex	0.024
glcm_DifferenceEntropy_Apex	-3.181807907
glcm_Imc2_Apex	-24.38850896
glcm_InverseVariance_Apex	-4.997607902
glcm_JointAverage_Apex	0.257
glcm_MCC_Apex	-5.063286745

glcm_MaximumProbability_Apex	274.934
glcm_SumAverage_Apex	0.000565326
glcm_SumEntropy_Apex	8.527
glrlm_GrayLevelNonUniformityNormalized_Apex	29.468
glszm_LowGrayLevelZoneEmphasis_Apex	-74.12210353
glszm_SizeZoneNonUniformity_Apex	-0.001095124
glszm_SmallAreaEmphasis_Apex	-21.1662332
glszm_SmallAreaHighGrayLevelEmphasis_Apex	0.000109585
glszm_SmallAreaLowGrayLevelEmphasis_Apex	-646.1424857
ngtdm_Coarseness_Apex	-100.5948909
ngtdm_Complexity_Apex	-0.0004838
ngtdm_Contrast_Apex	-9.923565475
gldm_DependenceNonUniformityNormalized_Apex	-8.445707876
gldm_DependenceVariance_Apex	1.657
gldm_GrayLevelNonUniformity_Apex	0.002130354
gldm_LargeDependenceLowGrayLevelEmphasis_Apex	11.498
gldm_SmallDependenceLowGrayLevelEmphasis_Apex	1392.455
firstorder_10Percentile_Base	-0.04819649
firstorder_InterquartileRange_Base	0.023
firstorder_Kurtosis_Base	0.099
firstorder_Median_Base	0.093
firstorder_Minimum_Base	0.067
firstorder_Range_Base	-0.024171931
firstorder_RobustMeanAbsoluteDeviation_Base	0.005885974
firstorder_Skewness_Base	0.311
firstorder_TotalEnergy_Base	-5.06827E-08
firstorder_Variance_Base	-0.000494672
shape_Elongation_Base	7.267
shape_Flatness_Base	-7.148647697
shape_MajorAxisLength_Base	0.015
shape_Maximum2DDiameterColumn_Base	0.088
shape_Maximum2DDiameterRow_Base	-0.029136784
shape_Maximum2DDiameterSlice_Base	0.145
shape_Maximum3DDiameter_Base	-0.15695807
shape_MinorAxisLength_Base	-0.299508723
shape_Sphericity_Base	7.676
shape_SurfaceArea_Base	0.001355521
shape_SurfaceVolumeRatio_Base	-7.966227392
glcm_ClusterProminence_Base	2.78103E-06
glcm_ClusterShade_Base	0.000147965
glcm_Contrast_Base	0.024
glcm_Correlation_Base	-4.439963012
glcm_DifferenceVariance_Base	-0.091992981
glcm_Imc1_Base	7.974
glcm_InverseVariance_Base	3.67
glcm_JointEntropy_Base	1.319
glcm_MCC_Base	-4.742227434
glcm_MaximumProbability_Base	27.757
glcm_SumSquares_Base	7.24288E-07
glrlm_GrayLevelNonUniformity_Base	0.000624266
glrlm_GrayLevelNonUniformityNormalized_Base	162.796

glrlm_GrayLevelVariance_Base	0.024
glrlm_RunLengthNonUniformityNormalized_Base	-2.037792585
glrlm_RunPercentage_Base	-43.98514778
glszm_GrayLevelVariance_Base	0.002715081
glszm_HighGrayLevelZoneEmphasis_Base	-0.005185856
glszm_LargeAreaEmphasis_Base	0.001428885
glszm_LargeAreaHighGrayLevelEmphasis_Base	-5.14652E-06
glszm_LargeAreaLowGrayLevelEmphasis_Base	-0.024674203
glszm_SizeZoneNonUniformity_Base	-0.000780204
glszm_SizeZoneNonUniformityNormalized_Base	31.825
glszm_SmallAreaHighGrayLevelEmphasis_Base	-0.000885754
ngtdm_Coarseness_Base	-258.3717435
ngtdm_Complexity_Base	-0.000762292
ngtdm_Contrast_Base	-29.49518732
ngtdm_Strength_Base	0.014
gldm_DependenceEntropy_Base	11.801
gldm_DependenceNonUniformityNormalized_Base	0.814
gldm_DependenceVariance_Base	-1.555376005
gldm_GrayLevelVariance_Base	0.017
gldm_LargeDependenceHighGrayLevelEmphasis_Base	-4.40462E-05
gldm_SmallDependenceLowGrayLevelEmphasis_Base	-664.1445652

The Radiomics features named as: Feature type_Feature name_Extraction area. WP: whole prostate.

Table S3. The trained PROSTATEx - U-Net model intercept and coefficients.

Feature	Coefficient
Intercept	-321.976
firstorder_10Percentile_WP	-0.361109955
firstorder_InterquartileRange_WP	0.169
firstorder_Kurtosis_WP	2.423
firstorder_Minimum_WP	-0.156260053
firstorder_Range_WP	0.039
firstorder_RobustMeanAbsoluteDeviation_WP	1.557
firstorder_TotalEnergy_WP	2.22304E-08
firstorder_Variance_WP	-0.046085079
shape_Elongation_WP	1.968
shape_MajorAxisLength_WP	-0.250079909
shape_Maximum2DDiameterColumn_WP	-0.257196182
shape_Maximum2DDiameterRow_WP	-0.145420583
shape_Maximum2DDiameterSlice_WP	-0.01886947
shape_Maximum3DDiameter_WP	-0.130069698
shape_MeshVolume_WP	0.000120006
shape_MinorAxisLength_WP	0.396
glcm_ClusterProminence_WP	2.71164E-06
glcm_ClusterShade_WP	0.000646111
glcm_Correlation_WP	-30.71256449
glcm_Idmn_WP	76.422
glcm_Imc1_WP	64.115
glcm_Imc2_WP	-42.31670998
glcm_MCC_WP	16.528
glcm_MaximumProbability_WP	-1094.72248
glszm_GrayLevelNonUniformityNormalized_WP	780.396

glszm_SizeZoneNonUniformity_WP	0.000570052
glszm_SmallAreaLowGrayLevelEmphasis_WP	-2417.522262
glszm_ZoneEntropy_WP	-8.843609049
glszm_ZonePercentage_WP	-7.43438445
ngtdm_Coarseness_WP	8643.934
ngtdm_Complexity_WP	-0.001616607
ngtdm_Strength_WP	4.063
gldm_DependenceEntropy_WP	41.577
gldm_DependenceNonUniformityNormalized_WP	-1.37948949
gldm_LargeDependenceHighGrayLevelEmphasis_WP	-0.001317192
gldm_LargeDependenceLowGrayLevelEmphasis_WP	64.421
firstorder_90Percentile_Apex	-0.107347033
firstorder_InterquartileRange_Apex	0.329
firstorder_Kurtosis_Apex	0.336
firstorder_Maximum_Apex	-0.000548995
firstorder_Median_Apex	-0.13347353
firstorder_Minimum_Apex	-0.024977611
firstorder_Variance_Apex	0.002616362
shape_Elongation_Apex	5.729
shape_LeastAxisLength_Apex	-0.410394504
shape_MajorAxisLength_Apex	-0.359425987
shape_Maximum2DDiameterColumn_Apex	0.279
shape_Maximum2DDiameterRow_Apex	0.036
shape_Maximum2DDiameterSlice_Apex	0.207
shape_MinorAxisLength_Apex	-0.384513413
shape_Sphericity_Apex	3.807
shape_SurfaceVolumeRatio_Apex	-44.062383
glcm_ClusterShade_Apex	-0.000321166
glcm_Correlation_Apex	6.09
glcm_Idn_Apex	-15.19375392
glcm_Imc2_Apex	-5.01588654
glcm_JointAverage_Apex	1.1824E-05
glcm_JointEnergy_Apex	-3038.847586
glcm_JointEntropy_Apex	-19.36122323
glcm_MCC_Apex	-11.42593655
glcm_MaximumProbability_Apex	2787.13
glcm_SumAverage_Apex	0.503
glcm_SumEntropy_Apex	44.621
glrlm_GrayLevelNonUniformity_Apex	-0.004539868
glrlm_LongRunLowGrayLevelEmphasis_Apex	-2134.126787
glrlm_ShortRunLowGrayLevelEmphasis_Apex	-665.1488514
glszm_GrayLevelNonUniformityNormalized_Apex	-691.5931433
glszm_GrayLevelVariance_Apex	0.013
glszm_SizeZoneNonUniformity_Apex	-0.000650814
glszm_SizeZoneNonUniformityNormalized_Apex	-54.0932208
glszm_SmallAreaEmphasis_Apex	-35.20923677
glszm_SmallAreaLowGrayLevelEmphasis_Apex	2823.613
glszm_ZoneEntropy_Apex	2.863
glszm_ZonePercentage_Apex	59.685
glszm_ZoneVariance_Apex	-0.006606771
ngtdm_Coarseness_Apex	1438.473

ngtdm_Complexity_Apex	-0.000288665
ngtdm_Strength_Apex	-3.219797197
gldm_DependenceEntropy_Apex	-36.05944425
gldm_DependenceVariance_Apex	8.133
gldm_HighGrayLevelEmphasis_Apex	-0.008487508
gldm_LargeDependenceHighGrayLevelEmphasis_Apex	-0.00071812
gldm_LargeDependenceLowGrayLevelEmphasis_Apex	81.651
firstorder_10Percentile_Base	0.204
firstorder_90Percentile_Base	0.292
firstorder_Kurtosis_Base	0.345
firstorder_Maximum_Base	-0.175831002
firstorder_RobustMeanAbsoluteDeviation_Base	1.058
firstorder_Skewness_Base	2.1
firstorder_TotalEnergy_Base	-1.01867E-07
shape_Elongation_Base	19.307
shape_Flatness_Base	-2.189503192
shape_LeastAxisLength_Base	0.142
shape_MajorAxisLength_Base	0.191
shape_Maximum2DDiameterColumn_Base	0.198
shape_Maximum2DDiameterRow_Base	-0.052203795
shape_Maximum3DDiameter_Base	0.025
shape_MinorAxisLength_Base	-0.395836828
shape_Sphericity_Base	-42.1834581
shape_SurfaceArea_Base	-0.001978519
shape_SurfaceVolumeRatio_Base	-30.98683539
glcm_Autocorrelation_Base	0.02
glcm_ClusterProminence_Base	1.6374E-05
glcm_ClusterShade_Base	-0.000524944
glcm_ClusterTendency_Base	-0.033890673
glcm_Idm_Base	-78.12670175
glcm_Idmn_Base	423.115
glcm_JointEnergy_Base	181.847
glcm_MCC_Base	10.921
glcm_MaximumProbability_Base	-41.13260693
glcm_SumSquares_Base	-0.05457626
glrlm_GrayLevelNonUniformityNormalized_Base	-145.1853283
glrlm_RunVariance_Base	-39.09025331
glrlm_ShortRunLowGrayLevelEmphasis_Base	68.658
glszm_LargeAreaHighGrayLevelEmphasis_Base	2.40474E-05
glszm_LargeAreaLowGrayLevelEmphasis_Base	0.387
glszm_LowGrayLevelZoneEmphasis_Base	1340.043
glszm_SizeZoneNonUniformityNormalized_Base	-7.925389028
glszm_SmallAreaLowGrayLevelEmphasis_Base	-900.8285969
glszm_ZoneVariance_Base	0.015
ngtdm_Busyness_Base	1.77
ngtdm_Complexity_Base	0.000728423
ngtdm_Strength_Base	-1.767239323
gldm_GrayLevelVariance_Base	-0.072416517
gldm_LargeDependenceLowGrayLevelEmphasis_Base	-64.18091988
gldm_SmallDependenceHighGrayLevelEmphasis_Base	-0.052635022

The Radiomics features named as: Feature type_Feature name_Extraction area. WP: whole prostate.

Table S4. The trained PROSTATEx - V-Net model intercept and coefficients.

Feature	Coefficient
Intercept	382.283
firstorder_90Percentile_WP	0.104
firstorder_InterquartileRange_WP	-0.12899759
firstorder_Median_WP	-0.184649075
firstorder_Minimum_WP	-0.258722838
firstorder_Skewness_WP	-0.499665432
firstorder_TotalEnergy_WP	1.14172E-08
firstorder_Uniformity_WP	734.318
firstorder_Variance_WP	-0.003975761
shape_Elongation_WP	14.085
shape_Flatness_WP	-26.24663162
shape_LeastAxisLength_WP	-0.188759158
shape_Maximum2DDiameterColumn_WP	-0.350192943
shape_Maximum2DDiameterRow_WP	-0.275736401
shape_Maximum2DDiameterSlice_WP	0.178
shape_Maximum3DDiameter_WP	0.103
shape_Sphericity_WP	23.296
shape_SurfaceVolumeRatio_WP	-187.1852196
glcm_Autocorrelation_WP	0.026
glcm_ClusterProminence_WP	-2.64673E-06
glcm_ClusterShade_WP	-0.000443921
glcm_Correlation_WP	-7.74557482
glcm_DifferenceVariance_WP	-0.205850712
glcm_Imc1_WP	49.886
glcm_MCC_WP	36.646
glcm_MaximumProbability_WP	572.011
glrlm_GrayLevelNonUniformity_WP	-0.001540021
glrlm_RunVariance_WP	-80.71373963
glszm_LargeAreaLowGrayLevelEmphasis_WP	0.032
glszm_SizeZoneNonUniformity_WP	0.000386128
glszm_SizeZoneNonUniformityNormalized_WP	-4.383540801
glszm_SmallAreaHighGrayLevelEmphasis_WP	0.019
glszm_SmallAreaLowGrayLevelEmphasis_WP	0.022
glszm_ZoneEntropy_WP	0.207
ngtdm_Coarseness_WP	31851.18
ngtdm_Complexity_WP	-0.000796818
ngtdm_Strength_WP	7.652
gldm_DependenceEntropy_WP	-3.524423106
gldm_LowGrayLevelEmphasis_WP	202.878
gldm_SmallDependenceLowGrayLevelEmphasis_WP	2828.786
firstorder_10Percentile_Apex	-0.176567555
firstorder_InterquartileRange_Apex	-0.139750995
firstorder_Kurtosis_Apex	-0.269244183
firstorder_Minimum_Apex	0.088
firstorder_Skewness_Apex	7.323
firstorder_Variance_Apex	0.011
shape_Elongation_Apex	-15.28827643
shape_Flatness_Apex	-9.750282188
shape_MajorAxisLength_Apex	-1.049820093

shape_Maximum2DDiameterColumn_Apex	-0.062538105
shape_Maximum2DDiameterRow_Apex	-0.011840197
shape_Maximum2DDiameterSlice_Apex	0.802
shape_Maximum3DDiameter_Apex	-0.797852217
shape_MeshVolume_Apex	0.000260848
shape_MinorAxisLength_Apex	0.241
shape_Sphericity_Apex	-69.6374527
shape_VoxelVolume_Apex	0.000124394
glcm_ClusterProminence_Apex	1.57165E-05
glcm_ClusterShade_Apex	-0.002219026
glcm_Correlation_Apex	12.85
glcm_DifferenceEntropy_Apex	-4.09303929
glcm_DifferenceVariance_Apex	-0.032803925
glcm_Imc1_Apex	-11.12989885
glcm_Imc2_Apex	-13.6901273
glcm_JointEnergy_Apex	-6990.37425
glcm_MCC_Apex	-31.9032659
glcm_MaximumProbability_Apex	1533.047
glcm_SumAverage_Apex	0.089
glcm_SumEntropy_Apex	13.538
glrlm_RunLengthNonUniformityNormalized_Apex	-92.79630735
glrlm_ShortRunEmphasis_Apex	-61.55064738
glszm_GrayLevelNonUniformityNormalized_Apex	101.119
glszm_LargeAreaHighGrayLevelEmphasis_Apex	-0.000181693
glszm_LargeAreaLowGrayLevelEmphasis_Apex	5.608
glszm_SmallAreaHighGrayLevelEmphasis_Apex	-0.014196094
glszm_ZoneVariance_Apex	-0.049201918
ngtdm_Busyness_Apex	2.637
ngtdm_Complexity_Apex	0.000669415
ngtdm_Contrast_Apex	-4.427026509
gldm_DependenceEntropy_Apex	1.031
gldm_DependenceNonUniformityNormalized_Apex	92.378
gldm_LargeDependenceEmphasis_Apex	3.355
gldm_LargeDependenceHighGrayLevelEmphasis_Apex	0.001035733
gldm_LargeDependenceLowGrayLevelEmphasis_Apex	-50.79463837
gldm_LowGrayLevelEmphasis_Apex	-157.2508035
gldm_SmallDependenceLowGrayLevelEmphasis_Apex	-2099.038966
firstorder_10Percentile_Base	-0.587515943
firstorder_90Percentile_Base	0.286
firstorder_Energy_Base	9.70197E-09
firstorder_InterquartileRange_Base	-0.393165233
firstorder_Kurtosis_Base	1.857
firstorder_Skewness_Base	-9.12033662
firstorder_TotalEnergy_Base	2.81459E-08
shape_Elongation_Base	18.966
shape_LeastAxisLength_Base	-0.177479526
shape_MajorAxisLength_Base	0.15
shape_Maximum2DDiameterColumn_Base	0.125
shape_Maximum2DDiameterRow_Base	0.037
shape_Maximum3DDiameter_Base	-0.245084468
shape_MinorAxisLength_Base	-0.658126709

shape_Sphericity_Base	-19.69112292
shape_SurfaceArea_Base	7.8765E-05
shape_SurfaceVolumeRatio_Base	-11.58048961
glcm_ClusterShade_Base	-0.000434701
glcm_Correlation_Base	1.614
glcm_DifferenceVariance_Base	-0.4222514
glcm_Idm_Base	-50.69111048
glcm_Imc2_Base	54.678
glcm_JointEnergy_Base	451.315
glcm_MCC_Base	-8.432719431
glcm_MaximumProbability_Base	1006.898
glcm_SumEntropy_Base	-5.542663873
glrlm_RunLengthNonUniformityNormalized_Base	-0.493380462
glrlm_RunVariance_Base	-37.80154633
glszm_GrayLevelNonUniformity_Base	-0.000570521
glszm_GrayLevelNonUniformityNormalized_Base	-717.5100996
glszm_LargeAreaEmphasis_Base	0.005691618
glszm_LargeAreaLowGrayLevelEmphasis_Base	0.003134206
glszm_LowGrayLevelZoneEmphasis_Base	-2803.662984
glszm_SmallAreaHighGrayLevelEmphasis_Base	-0.053575609
glszm_ZoneVariance_Base	3.25766E-07
ngtdm_Coarseness_Base	-13159.97686
ngtdm_Complexity_Base	0.001141479
ngtdm_Contrast_Base	58.232
gldm_DependenceEntropy_Base	-6.324520776
gldm_DependenceNonUniformityNormalized_Base	112.956
gldm_GrayLevelVariance_Base	0.055
gldm_SmallDependenceHighGrayLevelEmphasis_Base	-0.044455531

The Radiomics features named as: Feature type_Feature name_Extraction area. WP: whole prostate.

Table S5. The trained PROSTATEx - nnU-Net-2D model intercept and coefficients.

Feature	Coefficient
Intercept	796.981
firstorder_10Percentile_WP	0.29
firstorder_Energy_WP	1.19916E-10
firstorder_InterquartileRange_WP	0.636
firstorder_Maximum_WP	-0.109250329
firstorder_Minimum_WP	-0.694445779
shape_Elongation_WP	-24.70301357
shape_Flatness_WP	-14.17148864
shape_MajorAxisLength_WP	-0.718393217
shape_Maximum2DDiameterColumn_WP	-0.202531101
shape_Maximum2DDiameterRow_WP	-0.122970008
shape_Maximum2DDiameterSlice_WP	0.092
shape_Maximum3DDiameter_WP	-0.208910964
shape_SurfaceArea_WP	0.00044424
shape_SurfaceVolumeRatio_WP	-193.03525
glcm_ClusterProminence_WP	2.61885E-05
glcm_ClusterShade_WP	0.000594259
glcm_Correlation_WP	5.482
glcm_DifferenceVariance_WP	-0.236450592

glcm_JointEnergy_WP	866.556
glcm_MCC_WP	-5.194276409
glcm_SumSquares_WP	-0.085176649
glrlm_GrayLevelNonUniformity_WP	0.001755906
glrlm_GrayLevelNonUniformityNormalized_WP	162.861
glszm_GrayLevelNonUniformityNormalized_WP	-933.9826575
glszm_GrayLevelVariance_WP	0.026
glszm_HighGrayLevelZoneEmphasis_WP	0.001439849
glszm_SizeZoneNonUniformity_WP	0.000331595
glszm_SmallAreaLowGrayLevelEmphasis_WP	-1675.145157
glszm_ZoneEntropy_WP	-3.125394281
ngtdm_Coarseness_WP	2096.677
ngtdm_Complexity_WP	-0.000475403
ngtdm_Contrast_WP	-121.8550695
gldm_DependenceEntropy_WP	-15.70114408
gldm_DependenceNonUniformityNormalized_WP	178.047
gldm_LargeDependenceLowGrayLevelEmphasis_WP	19.94
gldm_LowGrayLevelEmphasis_WP	2135.631
firstorder_Kurtosis_Apex	-2.711844451
firstorder_Minimum_Apex	0.129
firstorder_Range_Apex	-0.07191723
firstorder_RobustMeanAbsoluteDeviation_Apex	-0.1420014
firstorder_Skewness_Apex	6.353
firstorder_TotalEnergy_Apex	4.13619E-08
shape_Elongation_Apex	3.668
shape_Flatness_Apex	-7.912707405
shape_LeastAxisLength_Apex	-0.10926489
shape_MajorAxisLength_Apex	-0.43237436
shape_Maximum2DDiameterColumn_Apex	0.189
shape_Maximum2DDiameterRow_Apex	-0.073192134
shape_Maximum2DDiameterSlice_Apex	0.178
shape_Maximum3DDiameter_Apex	-0.187472525
shape_SurfaceArea_Apex	0.000530272
glcm_ClusterProminence_Apex	2.77501E-05
glcm_ClusterShade_Apex	-0.000451384
glcm_Contrast_Apex	0.254
glcm_Correlation_Apex	-1.619781061
glcm_DifferenceEntropy_Apex	-17.07934278
glcm_DifferenceVariance_Apex	0.143
glcm_Imc2_Apex	-23.14455426
glcm_JointEnergy_Apex	-6212.219438
glcm_JointEntropy_Apex	-6.518581485
glcm_MaximumProbability_Apex	-120.8394487
glrlm_GrayLevelVariance_Apex	-0.036884918
glszm_GrayLevelVariance_Apex	-0.179393287
glszm_HighGrayLevelZoneEmphasis_Apex	0.021
glszm_LowGrayLevelZoneEmphasis_Apex	-267.8410649
glszm_SizeZoneNonUniformity_Apex	-0.001044345
glszm_ZoneVariance_Apex	-0.006817436
ngtdm_Complexity_Apex	-0.001420348
ngtdm_Contrast_Apex	-17.76628766

gldm_DependenceNonUniformityNormalized_Apex	-104.3249813
gldm_GrayLevelNonUniformity_Apex	-0.002647112
gldm_LargeDependenceLowGrayLevelEmphasis_Apex	0.441
gldm_SmallDependenceLowGrayLevelEmphasis_Apex	3108.422
firstorder_Energy_Base	2.51608E-08
firstorder_Entropy_Base	-2.704729568
firstorder_Kurtosis_Base	-0.419054007
firstorder_Skewness_Base	1.567
firstorder_Uniformity_Base	82.544
firstorder_Variance_Base	0.001343718
shape_Elongation_Base	-5.484035842
shape_Flatness_Base	-18.17738825
shape_LeastAxisLength_Base	0.996
shape_MajorAxisLength_Base	-0.311083432
shape_Maximum2DDiameterColumn_Base	0.425
shape_Maximum2DDiameterRow_Base	0.102
shape_Maximum2DDiameterSlice_Base	-0.375341977
shape_Maximum3DDiameter_Base	0.353
shape_SurfaceArea_Base	-0.001191261
shape_SurfaceVolumeRatio_Base	19.444
glcm_ClusterProminence_Base	-2.03688E-06
glcm_Correlation_Base	-5.390434191
glcm_InverseVariance_Base	37.919
glcm_JointAverage_Base	1.506
glcm_JointEntropy_Base	3.562
glcm_SumAverage_Base	0.001159842
glrlm_GrayLevelNonUniformityNormalized_Base	128.364
glrlm_RunLengthNonUniformityNormalized_Base	-388.4055959
glszm_LargeAreaHighGrayLevelEmphasis_Base	-3.98377E-05
glszm_LowGrayLevelZoneEmphasis_Base	-749.5529367
glszm_SmallAreaHighGrayLevelEmphasis_Base	-0.007953134
glszm_ZoneEntropy_Base	2.794
ngtdm_Busyness_Base	-4.202338416
ngtdm_Complexity_Base	-0.001070331
ngtdm_Contrast_Base	23.444
ngtdm_Strength_Base	-1.538993176
gldm_DependenceNonUniformityNormalized_Base	135.232
gldm_HighGrayLevelEmphasis_Base	-0.01486939
gldm_LargeDependenceEmphasis_Base	0.262
gldm_LargeDependenceHighGrayLevelEmphasis_Base	-0.001878785
gldm_LargeDependenceLowGrayLevelEmphasis_Base	-38.97542234
gldm_SmallDependenceLowGrayLevelEmphasis_Base	4057.745

The Radiomics features named as: Feature type_Feature name_Extraction area. WP: whole prostate.

Table S6. The trained PROSTATEx - nnU-Net-3D model intercept and coefficients.

Feature	Coefficient
Intercept	1309.623
firstorder_10Percentile_WP	-0.721142033
firstorder_Energy_WP	1.11777E-08
firstorder_InterquartileRange_WP	0.466
firstorder_Kurtosis_WP	-1.807098348

firstorder_Minimum_WP	-0.414416705
firstorder_RobustMeanAbsoluteDeviation_WP	0.343
firstorder_Skewness_WP	2.235
firstorder_TotalEnergy_WP	2.76743E-08
shape_Elongation_WP	-14.90949407
shape_Flatness_WP	5.777
shape_Maximum2DDiameterColumn_WP	-0.02988986
shape_Maximum3DDiameter_WP	-0.108640637
shape_MeshVolume_WP	0.000129798
shape_SurfaceArea_WP	-0.005093056
shape_SurfaceVolumeRatio_WP	-47.40701402
shape_VoxelVolume_WP	3.04889E-05
glcm_ClusterShade_WP	0.001115036
glcm_Contrast_WP	-0.362129542
glcm_DifferenceVariance_WP	-0.116939892
glcm_Imc1_WP	117.02
glcm_JointAverage_WP	0.944
glcm_MCC_WP	-10.81140584
glcm_MaximumProbability_WP	50.582
glcm_SumAverage_WP	0.045
glrlm_GrayLevelNonUniformity_WP	0.001113064
glrlm_GrayLevelVariance_WP	-0.25316124
glszm_GrayLevelVariance_WP	-0.023530978
glszm_LargeAreaHighGrayLevelEmphasis_WP	-1.76798E-06
glszm_LowGrayLevelZoneEmphasis_WP	2739.237
glszm_SmallAreaEmphasis_WP	82.808
glszm_SmallAreaHighGrayLevelEmphasis_WP	0.021
ngtdm_Busyness_WP	1.076
ngtdm_Coarseness_WP	-0.939921946
ngtdm_Complexity_WP	0.00427186
ngtdm_Strength_WP	-4.814057849
gldm_DependenceVariance_WP	-2.537048333
gldm_SmallDependenceLowGrayLevelEmphasis_WP	-4212.588098
firstorder_10Percentile_Apex	-0.466481814
firstorder_InterquartileRange_Apex	-0.114658415
firstorder_Kurtosis_Apex	-1.75226954
firstorder_Median_Apex	0.33
firstorder_Minimum_Apex	0.395
firstorder_Range_Apex	-0.065000378
firstorder_Skewness_Apex	8.473
shape_Elongation_Apex	1.819
shape_Flatness_Apex	-25.91600339
shape_LeastAxisLength_Apex	0.661
shape_MajorAxisLength_Apex	-0.439242302
shape_Maximum2DDiameterColumn_Apex	0.07
shape_Maximum2DDiameterRow_Apex	-0.177117185
shape_Maximum2DDiameterSlice_Apex	0.144
shape_Maximum3DDiameter_Apex	-0.068137016
glcm_ClusterProminence_Apex	1.65745E-05
glcm_ClusterShade_Apex	-0.000651419
glcm_Correlation_Apex	-28.7185583

glcm_DifferenceVariance_Apex	-0.124905076
glcm_Imc1_Apex	-38.63027152
glcm_Imc2_Apex	3.105
glcm_InverseVariance_Apex	-222.5726206
glcm_JointEnergy_Apex	615.764
glcm_MCC_Apex	1.043
glrlm_LongRunLowGrayLevelEmphasis_Apex	370.289
glrlm_ShortRunEmphasis_Apex	-364.7467476
glszm_GrayLevelVariance_Apex	0.014
glszm_LargeAreaHighGrayLevelEmphasis_Apex	-0.000126603
glszm_SmallAreaEmphasis_Apex	-61.04673102
glszm_SmallAreaLowGrayLevelEmphasis_Apex	-1246.855983
ngtdm_Busyness_Apex	0.111
ngtdm_Complexity_Apex	-0.001052067
ngtdm_Contrast_Apex	2.963
gldm_DependenceEntropy_Apex	5.341
gldm_DependenceNonUniformity_Apex	-0.000773742
gldm_DependenceVariance_Apex	4.539
gldm_LargeDependenceHighGrayLevelEmphasis_Apex	0.00149014
gldm_SmallDependenceHighGrayLevelEmphasis_Apex	-0.00868048
firstorder_10Percentile_Base	1.494
firstorder_InterquartileRange_Base	-0.297270611
firstorder_Kurtosis_Base	0.841
firstorder_Median_Base	-1.129276822
firstorder_TotalEnergy_Base	9.28406E-09
firstorder_Uniformity_Base	-967.8388438
firstorder_Variance_Base	0.016
shape_Elongation_Base	-11.1824233
shape_Flatness_Base	-1.92091796
shape_MajorAxisLength_Base	-0.07769645
shape_Maximum2DDiameterColumn_Base	0.035
shape_Maximum2DDiameterRow_Base	0.177
shape_Maximum2DDiameterSlice_Base	0.333
shape_Maximum3DDiameter_Base	-0.454223481
shape_MinorAxisLength_Base	-0.041608717
shape_Sphericity_Base	-54.66939797
shape_SurfaceVolumeRatio_Base	-48.9220416
glcm_ClusterShade_Base	-0.0007041
glcm_ClusterTendency_Base	-0.003726705
glcm_Correlation_Base	27.166
glcm_DifferenceVariance_Base	0.191
glcm_Imc1_Base	16.911
glcm_JointAverage_Base	2.628
glcm_MCC_Base	5.967
glcm_MaximumProbability_Base	-591.1130396
glcm_SumEntropy_Base	-26.76138121
glrlm_RunLengthNonUniformity_Base	-0.000192395
glrlm_RunLengthNonUniformityNormalized_Base	-675.9008458
glrlm_RunVariance_Base	-172.7504116
glszm_GrayLevelVariance_Base	3.21173E-06
glszm_LargeAreaLowGrayLevelEmphasis_Base	1.932

glszm_SizeZoneNonUniformityNormalized_Base	71.564
glszm_SmallAreaHighGrayLevelEmphasis_Base	-0.040155764
glszm_SmallAreaLowGrayLevelEmphasis_Base	139.748
glszm_ZoneEntropy_Base	4.103
ngtdm_Busyness_Base	1.303
ngtdm_Coarseness_Base	-364.9687301
ngtdm_Complexity_Base	-0.000170833
ngtdm_Contrast_Base	49.398
ngtdm_Strength_Base	2.141
gldm_DependenceNonUniformity_Base	-0.00106838
gldm_DependenceVariance_Base	-6.512468818
gldm_LargeDependenceHighGrayLevelEmphasis_Base	-0.002266611
gldm_SmallDependenceLowGrayLevelEmphasis_Base	263.478

The Radiomics features named as: Feature type_Feature name_Extraction area. WP: whole prostate.

Table S7. The trained In-house - U-Net model intercept and coefficients.

Feature	Coefficient
Intercept	58.793
firstorder_10Percentile_WP	-0.409826323
firstorder_InterquartileRange_WP	0.105
firstorder_Kurtosis_WP	0.115
firstorder_Minimum_WP	0.642
firstorder_Range_WP	-0.010042831
firstorder_Skewness_WP	-1.126762793
firstorder_Variance_WP	0.021
shape_Elongation_WP	5.546
shape_LeastAxisLength_WP	-0.588233617
shape_MajorAxisLength_WP	-0.009761257
shape_Maximum2DDiameterColumn_WP	-0.287024615
shape_Maximum2DDiameterRow_WP	-0.084692949
shape_Maximum3DDiameter_WP	-0.35243464
shape_MeshVolume_WP	0.000124765
shape_Sphericity_WP	48.425
shape_SurfaceVolumeRatio_WP	75.795
shape_VoxelVolume_WP	0.0001127
glcm_Autocorrelation_WP	0.065
glcm_ClusterShade_WP	-0.000371266
glcm_ClusterTendency_WP	-0.039895043
glcm_JointAverage_WP	0.00043459
glcm_JointEnergy_WP	-3086.280781
glcm_MCC_WP	-0.682031328
glcm_MaximumProbability_WP	1315.363
glcm_SumAverage_WP	0.011
glcm_SumSquares_WP	-0.209410018
glrlm_RunEntropy_WP	-2.523579447
glszm_GrayLevelNonUniformityNormalized_WP	-1786.604709
glszm_LargeAreaHighGrayLevelEmphasis_WP	-4.08143E-07
glszm_LargeAreaLowGrayLevelEmphasis_WP	-0.076981146
glszm_LowGrayLevelZoneEmphasis_WP	169.134
glszm_SmallAreaHighGrayLevelEmphasis_WP	-0.05355589
glszm_SmallAreaLowGrayLevelEmphasis_WP	120.991

glszm_ZoneEntropy_WP	-2.334812754
glszm_ZonePercentage_WP	-24.33033128
ngtdm_Busyness_WP	1.616
ngtdm_Coarseness_WP	-31168.57073
ngtdm_Complexity_WP	-0.000218006
ngtdm_Contrast_WP	17.704
gldm_DependenceNonUniformity_WP	0.000725363
gldm_DependenceNonUniformityNormalized_WP	-202.4339009
gldm_DependenceVariance_WP	-5.690421242
gldm_LargeDependenceHighGrayLevelEmphasis_WP	-0.001691745
gldm_SmallDependenceLowGrayLevelEmphasis_WP	-2640.112347
firstorder_10Percentile_Apex	1.024
firstorder_Energy_Apex	-5.20773E-08
firstorder_Kurtosis_Apex	-2.783838802
firstorder_Maximum_Apex	-0.152991221
firstorder_Minimum_Apex	-0.078605903
firstorder_Skewness_Apex	4.895
shape_Elongation_Apex	-23.91431753
shape_Flatness_Apex	44.268
shape_LeastAxisLength_Apex	-1.042189536
shape_MajorAxisLength_Apex	-0.180133877
shape_Maximum2DDiameterColumn_Apex	0.037
shape_Maximum3DDiameter_Apex	0.162
shape_MinorAxisLength_Apex	0.638
shape_Sphericity_Apex	-1.663816303
shape_SurfaceArea_Apex	-9.66892E-05
shape_SurfaceVolumeRatio_Apex	-3.74923741
glcm_ClusterProminence_Apex	1.20644E-05
glcm_Contrast_Apex	0.237
glcm_Correlation_Apex	3.756
glcm_Idm_Apex	94.084
glcm_Imc1_Apex	191.307
glcm_Imc2_Apex	-19.80854251
glcm_JointAverage_Apex	-0.165133348
glcm_MCC_Apex	4.809
glcm_MaximumProbability_Apex	-592.7530633
glcm_SumAverage_Apex	-0.164328998
glrlm_RunVariance_Apex	255.402
glszm_GrayLevelNonUniformity_Apex	-0.007447548
glszm_GrayLevelNonUniformityNormalized_Apex	-335.9868087
glszm_GrayLevelVariance_Apex	0.054
glszm_LargeAreaEmphasis_Apex	0.006333208
glszm_SmallAreaLowGrayLevelEmphasis_Apex	-582.8753445
glszm_ZoneEntropy_Apex	32.543
ngtdm_Busyness_Apex	-4.009037279
ngtdm_Complexity_Apex	-0.001872825
ngtdm_Contrast_Apex	-76.42068449
ngtdm_Strength_Apex	2.212
gldm_DependenceEntropy_Apex	-11.90863505
gldm_DependenceNonUniformityNormalized_Apex	93.417
gldm_LargeDependenceEmphasis_Apex	1.168

gldm_LargeDependenceHighGrayLevelEmphasis_Apex	-0.001201862
gldm_LargeDependenceLowGrayLevelEmphasis_Apex	-9.513155846
gldm_LowGrayLevelEmphasis_Apex	1653.482
firstorder_90Percentile_Base	-0.156919732
firstorder_Energy_Base	-5.6035E-08
firstorder_InterquartileRange_Base	-0.040518043
firstorder_Kurtosis_Base	1.093
firstorder_Maximum_Base	-0.042287615
firstorder_Minimum_Base	-0.174962769
firstorder_Skewness_Base	-4.199818513
firstorder_Uniformity_Base	-218.3511658
firstorder_Variance_Base	0.012
shape_Elongation_Base	-27.82042158
shape_Flatness_Base	33.905
shape_LeastAxisLength_Base	-0.561529979
shape_MajorAxisLength_Base	0.014
shape_Maximum2DDiameterColumn_Base	-0.164212242
shape_Maximum2DDiameterRow_Base	-0.122066823
shape_Maximum2DDiameterSlice_Base	0.164
shape_Maximum3DDiameter_Base	-0.012223014
shape_MinorAxisLength_Base	0.532
shape_Sphericity_Base	25.803
shape_SurfaceArea_Base	0.002999379
shape_SurfaceVolumeRatio_Base	-26.29355783
glcm_ClusterProminence_Base	9.642E-06
glcm_ClusterShade_Base	-0.000269434
glcm_Contrast_Base	0.045
glcm_Correlation_Base	-20.08108148
glcm_DifferenceEntropy_Base	-11.68204478
glcm_DifferenceVariance_Base	-0.164338494
glcm_Id_Base	-30.79271011
glcm_Idm_Base	-60.74166585
glcm_Imc1_Base	63.589
glcm_Imc2_Base	31.805
glcm_InverseVariance_Base	90.95
glcm_JointAverage_Base	-0.251969859
glcm_JointEnergy_Base	3.817
glcm_JointEntropy_Base	1.812
glcm_MCC_Base	-25.5010576
glcm_SumAverage_Base	-0.005675909
glcm_SumSquares_Base	0.15
glrlm_LongRunEmphasis_Base	-19.93426572
glszm_GrayLevelVariance_Base	0.066
glszm_LargeAreaHighGrayLevelEmphasis_Base	3.3941E-05
glszm_LargeAreaLowGrayLevelEmphasis_Base	-0.024327213
glszm_LowGrayLevelZoneEmphasis_Base	-139.2555514
glszm_SmallAreaEmphasis_Base	44.033
glszm_ZoneEntropy_Base	13.789
ngtdm_Busyness_Base	0.17
ngtdm_Coarseness_Base	1130.563
ngtdm_Complexity_Base	-0.001859765

ngtdm_Contrast_Base	-20.48422515
ngtdm_Strength_Base	0.192
gldm_DependenceNonUniformity_Base	-0.003340815
gldm_DependenceNonUniformityNormalized_Base	57.677
gldm_DependenceVariance_Base	1.992
gldm_GrayLevelNonUniformity_Base	-0.002678311
gldm_GrayLevelVariance_Base	-0.16360058
gldm_LargeDependenceHighGrayLevelEmphasis_Base	-0.001055181
gldm_LargeDependenceLowGrayLevelEmphasis_Base	6.17
gldm_SmallDependenceLowGrayLevelEmphasis_Base	-1014.191344

The Radiomics features named as: Feature type_Feature name_Extraction area. WP: whole prostate.

Table S8. The trained In-house - V-Net model intercept and coefficients.

Feature	Coefficient
Intercept	-665.676
firstorder_10Percentile_WP	0.607
firstorder_Energy_WP	-2.47051E-08
firstorder_Kurtosis_WP	-3.202042156
firstorder_MeanAbsoluteDeviation_WP	0.823
firstorder_Minimum_WP	-0.060290664
firstorder_Skewness_WP	6.37
firstorder_Uniformity_WP	964.467
firstorder_Variance_WP	0.022
shape_Elongation_WP	2.541
shape_Flatness_WP	-11.00444747
shape_LeastAxisLength_WP	-0.185191432
shape_MajorAxisLength_WP	-1.024911096
shape_Maximum2DDiameterColumn_WP	0.217
shape_Maximum2DDiameterRow_WP	0.035
shape_Maximum3DDiameter_WP	-0.090905338
shape_Sphericity_WP	161.442
glcm_ClusterShade_WP	-0.003112853
glcm_DifferenceVariance_WP	-0.185309313
glcm_JointAverage_WP	0.219
glcm_MCC_WP	37.95
glcm_MaximumProbability_WP	867.141
glcm_SumAverage_WP	0.008326982
glrlm_GrayLevelNonUniformityNormalized_WP	812.002
glszm_GrayLevelVariance_WP	-0.001948064
glszm_LargeAreaHighGrayLevelEmphasis_WP	-7.69233E-06
glszm_LargeAreaLowGrayLevelEmphasis_WP	0.033
glszm_LowGrayLevelZoneEmphasis_WP	-988.8339253
glszm_SizeZoneNonUniformity_WP	0.002356206
glszm_SizeZoneNonUniformityNormalized_WP	38.939
glszm_SmallAreaLowGrayLevelEmphasis_WP	-2355.458998
glszm_ZoneEntropy_WP	11.398
ngtdm_Busyness_WP	-0.169001974
ngtdm_Complexity_WP	0.002545098
ngtdm_Strength_WP	-4.096543889
gldm_DependenceEntropy_WP	20.5
gldm_DependenceVariance_WP	-1.415300226

gldm_GrayLevelNonUniformity_WP	-0.001548627
gldm_LargeDependenceLowGrayLevelEmphasis_WP	-22.42378295
gldm_SmallDependenceLowGrayLevelEmphasis_WP	9605.562
firstorder_Kurtosis_Apex	-1.468692815
firstorder_Mean_Apex	-0.102365267
firstorder_Minimum_Apex	1.103
firstorder_Range_Apex	-0.040394544
firstorder_TotalEnergy_Apex	8.33531E-09
firstorder_Uniformity_Apex	-94.07051406
shape_Flatness_Apex	12.813
shape_LeastAxisLength_Apex	-1.13644107
shape_MajorAxisLength_Apex	0.061
shape_Maximum2DDiameterColumn_Apex	0.058
shape_Maximum2DDiameterRow_Apex	-0.00670403
shape_Maximum2DDiameterSlice_Apex	-0.126244377
shape_Maximum3DDiameter_Apex	-0.170450804
shape_MinorAxisLength_Apex	-0.606954253
shape_Sphericity_Apex	-115.8931811
shape_SurfaceVolumeRatio_Apex	-47.23503452
glcm_ClusterProminence_Apex	-1.02783E-05
glcm_ClusterShade_Apex	0.000543035
glcm_Correlation_Apex	-5.541639536
glcm_Idm_Apex	-30.61394688
glcm_Imc1_Apex	76.203
glcm_MCC_Apex	10.11
glrlm_GrayLevelNonUniformity_Apex	0.008073803
glrlm_LongRunLowGrayLevelEmphasis_Apex	59.78
glszm_GrayLevelVariance_Apex	-0.022018761
glszm_HighGrayLevelZoneEmphasis_Apex	0.027
glszm_SizeZoneNonUniformity_Apex	-0.003308016
glszm_SizeZoneNonUniformityNormalized_Apex	-31.49718966
glszm_SmallAreaLowGrayLevelEmphasis_Apex	-980.0185665
ngtdm_Busyness_Apex	6.393
ngtdm_Coarseness_Apex	-12137.96129
ngtdm_Complexity_Apex	-0.000147367
ngtdm_Strength_Apex	7.118
gldm_GrayLevelNonUniformity_Apex	0.007816823
gldm_LargeDependenceHighGrayLevelEmphasis_Apex	-0.000363938
gldm_LargeDependenceLowGrayLevelEmphasis_Apex	17.551
firstorder_90Percentile_Base	0.311
firstorder_Energy_Base	-5.24322E-08
firstorder_InterquartileRange_Base	-0.409002608
firstorder_Maximum_Base	-0.15112896
firstorder_Minimum_Base	-0.358450791
firstorder_Skewness_Base	-0.554905249
shape_Elongation_Base	2.538
shape_Flatness_Base	35.184
shape_LeastAxisLength_Base	-0.375346084
shape_MajorAxisLength_Base	0.373
shape_Maximum2DDiameterColumn_Base	-0.204313436
shape_Maximum2DDiameterRow_Base	-0.418496704

shape_Maximum2DDiameterSlice_Base	0.048
shape_Maximum3DDiameter_Base	0.025
shape_Sphericity_Base	-39.96665213
shape_SurfaceArea_Base	0.003922066
shape_SurfaceVolumeRatio_Base	-14.51836726
glcm_ClusterProminence_Base	4.57063E-06
glcm_ClusterTendency_Base	0.012
glcm_Correlation_Base	-5.153969245
glcm_DifferenceVariance_Base	0.176
glcm_Idn_Base	404.812
glcm_Imc1_Base	-6.685165756
glcm_Imc2_Base	1.258
glcm_JointAverage_Base	0.925
glcm_JointEnergy_Base	-1718.336606
glcm_MCC_Base	2.527
glcm_MaximumProbability_Base	-401.0091738
glrlm_GrayLevelNonUniformity_Base	0.004031616
glrlm_GrayLevelNonUniformityNormalized_Base	99.731
glrlm_ShortRunLowGrayLevelEmphasis_Base	366.209
glszm_GrayLevelNonUniformity_Base	0.00597847
glszm_HighGrayLevelZoneEmphasis_Base	-0.04705148
glszm_LargeAreaEmphasis_Base	0.002089843
glszm_LargeAreaHighGrayLevelEmphasis_Base	-1.61651E-05
glszm_LowGrayLevelZoneEmphasis_Base	36.704
glszm_SmallAreaEmphasis_Base	98.631
glszm_ZoneVariance_Base	0.003766068
ngtdm_Busyness_Base	0.447
ngtdm_Complexity_Base	-0.00019638
ngtdm_Strength_Base	1.893
gldm_DependenceNonUniformityNormalized_Base	-43.49194441
gldm_LargeDependenceHighGrayLevelEmphasis_Base	0.000813639
gldm_SmallDependenceLowGrayLevelEmphasis_Base	-1314.284524

The Radiomics features named as: Feature type_Feature name_Extraction area. WP: whole prostate.

Table S9. The trained In-house - nnU-Net-2D model intercept and coefficients.

Feature	Coefficient
Intercept	-75.803
firstorder_10Percentile_WP	-0.952585663
firstorder_InterquartileRange_WP	-0.804508418
firstorder_Kurtosis_WP	1.149
firstorder_Maximum_WP	0.239
firstorder_Minimum_WP	1.595
firstorder_Skewness_WP	-5.171647096
firstorder_Variance_WP	0.016
shape_Elongation_WP	-14.52052431
shape_Flatness_WP	-9.36098304
shape_MajorAxisLength_WP	-0.448750298
shape_Maximum2DDiameterColumn_WP	-0.355542567
shape_Maximum2DDiameterRow_WP	-0.230861438
shape_Maximum2DDiameterSlice_WP	-0.13734098
shape_Sphericity_WP	80.22

shape_SurfaceArea_WP	0.00059422
shape_SurfaceVolumeRatio_WP	182.034
shape_VoxelVolume_WP	0.000197024
glcm_ClusterShade_WP	-0.0002725
glcm_Idmn_WP	133.637
glcm_Imc1_WP	-160.1112042
glcm_Imc2_WP	-52.75569003
glcm_InverseVariance_WP	-83.77687998
glcm_MCC_WP	-25.98528238
glcm_MaximumProbability_WP	104.238
glszm_GrayLevelVariance_WP	0.198
glszm_LargeAreaLowGrayLevelEmphasis_WP	0.082
glszm_LowGrayLevelZoneEmphasis_WP	-109.501208
glszm_SizeZoneNonUniformityNormalized_WP	2.242
ngtdm_Busyness_WP	-1.679498807
ngtdm_Complexity_WP	9.96145E-05
ngtdm_Contrast_WP	-58.94837564
ngtdm_Strength_WP	-40.34304987
gldm_DependenceNonUniformity_WP	0.001733947
gldm_LargeDependenceHighGrayLevelEmphasis_WP	0.002343983
gldm_LargeDependenceLowGrayLevelEmphasis_WP	32.69
firstorder_Energy_Apex	-2.25125E-08
firstorder_Kurtosis_Apex	-4.694483056
firstorder_Mean_Apex	0.081
firstorder_Median_Apex	0.2
firstorder_Minimum_Apex	0.026
firstorder_RobustMeanAbsoluteDeviation_Apex	-0.612443446
firstorder_Skewness_Apex	2.87
firstorder_Variance_Apex	-0.014627929
shape_Elongation_Apex	-1.486408625
shape_Flatness_Apex	-5.474652002
shape_LeastAxisLength_Apex	0.322
shape_MajorAxisLength_Apex	0.356
shape_Maximum2DDiameterColumn_Apex	0.062
shape_Maximum2DDiameterRow_Apex	-0.120393147
shape_Maximum2DDiameterSlice_Apex	-0.306718435
shape_Maximum3DDiameter_Apex	0.118
shape_Sphericity_Apex	-25.47544312
shape_SurfaceArea_Apex	-0.00115801
glcm_Autocorrelation_Apex	0.00212524
glcm_ClusterProminence_Apex	1.35505E-05
glcm_ClusterShade_Apex	0.00023352
glcm_Correlation_Apex	-31.78202127
glcm_DifferenceVariance_Apex	0.004458033
glcm_Imc2_Apex	-19.37831006
glcm_InverseVariance_Apex	-75.37312521
glcm_JointEnergy_Apex	-650.9100976
glcm_MCC_Apex	30.236
glcm_MaximumProbability_Apex	1369.891
glcm_SumEntropy_Apex	23.394
glrlm_GrayLevelNonUniformity_Apex	-0.008937945

glrlm_LongRunEmphasis_Apex	-78.29580954
glrlm_ShortRunEmphasis_Apex	340.148
glszm_GrayLevelNonUniformity_Apex	-3.15839E-07
glszm_LargeAreaHighGrayLevelEmphasis_Apex	8.70944E-05
glszm_LowGrayLevelZoneEmphasis_Apex	-892.9189737
glszm_SizeZoneNonUniformity_Apex	0.000268337
glszm_SmallAreaEmphasis_Apex	68.688
glszm_ZoneEntropy_Apex	-1.497017078
glszm_ZonePercentage_Apex	-298.2110028
ngtdm_Contrast_Apex	5.488
gldm_DependenceEntropy_Apex	-22.24853234
gldm_LargeDependenceEmphasis_Apex	-1.350096236
gldm_LargeDependenceHighGrayLevelEmphasis_Apex	-0.001367631
gldm_LowGrayLevelEmphasis_Apex	1109.321
firstorder_90Percentile_Base	0.038
firstorder_InterquartileRange_Base	0.056
firstorder_Maximum_Base	-0.068806763
firstorder_Median_Base	-0.167674187
firstorder_Minimum_Base	-0.108160733
firstorder_Skewness_Base	-3.71693444
firstorder_TotalEnergy_Base	4.37878E-08
firstorder_Variance_Base	0.009403888
shape_Elongation_Base	41.712
shape_Flatness_Base	-15.3874693
shape_LeastAxisLength_Base	0.022
shape_MajorAxisLength_Base	0.467
shape_Maximum2DDiameterColumn_Base	0.034
shape_Maximum2DDiameterRow_Base	-0.106613069
shape_Maximum2DDiameterSlice_Base	0.362
shape_MinorAxisLength_Base	-0.895357483
shape_Sphericity_Base	1.776
shape_SurfaceArea_Base	0.001371277
glcm_Autocorrelation_Base	-0.018914542
glcm_ClusterShade_Base	0.000367885
glcm_Correlation_Base	4.784
glcm_DifferenceVariance_Base	-0.13629786
glcm_Imc1_Base	36.779
glcm_Imc2_Base	-27.53258125
glcm_JointEnergy_Base	491.857
glcm_JointEntropy_Base	3.118
glcm_MCC_Base	10.865
glrlm_RunEntropy_Base	15.824
glrlm_RunLengthNonUniformity_Base	-0.000408762
glszm_HighGrayLevelZoneEmphasis_Base	0.026
glszm_LargeAreaEmphasis_Base	-0.034765993
glszm_SizeZoneNonUniformity_Base	-0.003023618
glszm_SmallAreaLowGrayLevelEmphasis_Base	1459.157
glszm_ZoneEntropy_Base	-15.84480995
glszm_ZonePercentage_Base	-79.29353486
ngtdm_Coarseness_Base	2348.112
ngtdm_Complexity_Base	0.001814881

ngtdm_Contrast_Base	31.051
gldm_GrayLevelVariance_Base	-0.159526151
gldm_LargeDependenceEmphasis_Base	-0.042181185
gldm_LowGrayLevelEmphasis_Base	-834.1902871
gldm_SmallDependenceLowGrayLevelEmphasis_Base	-408.9185167

The Radiomics features named as: Feature type_Feature name_Extraction area. WP: whole prostate.

Table S10. The trained In-house - nnU-Net-3D model intercept and coefficients.

Feature	Coefficient
Intercept	-149.82
firstorder_Energy_WP	-2.47222E-08
firstorder_InterquartileRange_WP	-0.106069147
firstorder_Kurtosis_WP	-1.647215782
firstorder_Mean_WP	1.313
firstorder_Minimum_WP	1.037
firstorder_Range_WP	-0.165230167
firstorder_Skewness_WP	25.027
shape_Elongation_WP	0.561
shape_Flatness_WP	12.073
shape_LeastAxisLength_WP	-0.288934047
shape_MajorAxisLength_WP	-0.02937799
shape_Maximum2DDiameterRow_WP	0.013
shape_Maximum2DDiameterSlice_WP	0.062
shape_Maximum3DDiameter_WP	-0.106485299
shape_MinorAxisLength_WP	0.123
shape_Sphericity_WP	100.511
glcm_ClusterShade_WP	-0.003130491
glcm_ClusterTendency_WP	0.04
glcm_Contrast_WP	-0.000125038
glcm_Correlation_WP	0.053
glcm_DifferenceVariance_WP	-0.198911163
glcm_Imc2_WP	-1.265234243
glcm_InverseVariance_WP	-20.32500725
glcm_MaximumProbability_WP	717.81
glrlm_ShortRunEmphasis_WP	345.005
glszm_GrayLevelNonUniformity_WP	0.009552618
glszm_LargeAreaHighGrayLevelEmphasis_WP	-2.47514E-06
glszm_LargeAreaLowGrayLevelEmphasis_WP	-0.387610541
glszm_LowGrayLevelZoneEmphasis_WP	-459.4574471
glszm_SizeZoneNonUniformity_WP	0.000138626
glszm_SmallAreaHighGrayLevelEmphasis_WP	-0.021041159
glszm_SmallAreaLowGrayLevelEmphasis_WP	-1052.318132
glszm_ZoneEntropy_WP	-12.53775801
ngtdm_Busyness_WP	1.568
ngtdm_Coarseness_WP	-26321.58435
ngtdm_Complexity_WP	-0.001319323
ngtdm_Strength_WP	14.672
gldm_DependenceEntropy_WP	18.6
gldm_LargeDependenceLowGrayLevelEmphasis_WP	69.318
firstorder_10Percentile_Apex	-0.157698579
firstorder_InterquartileRange_Apex	-0.299308216

firstorder_Kurtosis_Apex	-4.023784202
firstorder_Minimum_Apex	-0.86249131
firstorder_Range_Apex	0.109
firstorder_TotalEnergy_Apex	8.38876E-08
firstorder_Variance_Apex	-0.012439016
shape_Elongation_Apex	6.766
shape_LeastAxisLength_Apex	0.205
shape_MajorAxisLength_Apex	0.173
shape_Maximum2DDiameterColumn_Apex	0.068
shape_Maximum2DDiameterRow_Apex	-0.05706543
shape_Maximum2DDiameterSlice_Apex	-0.269497398
shape_Maximum3DDiameter_Apex	0.136
shape_Sphericity_Apex	0.508
shape_SurfaceArea_Apex	-0.00020118
shape_SurfaceVolumeRatio_Apex	-9.955860422
glcm_Autocorrelation_Apex	0.048
glcm_ClusterProminence_Apex	1.80532E-06
glcm_ClusterShade_Apex	0.000398695
glcm_Correlation_Apex	-21.41000414
glcm_DifferenceEntropy_Apex	-5.340768914
glcm_DifferenceVariance_Apex	-0.009513745
glcm_Idm_Apex	193.115
glcm_Imc2_Apex	-5.607008112
glcm_MCC_Apex	8.623
glcm_MaximumProbability_Apex	9.281
glcm_SumAverage_Apex	0.103
glrlm_ShortRunLowGrayLevelEmphasis_Apex	599.045
glszm_GrayLevelVariance_Apex	0.179
glszm_LargeAreaEmphasis_Apex	0.085
glszm_LargeAreaLowGrayLevelEmphasis_Apex	-0.671634403
glszm_SmallAreaEmphasis_Apex	-110.3198284
glszm_SmallAreaLowGrayLevelEmphasis_Apex	-217.001015
glszm_ZoneEntropy_Apex	-26.84580904
glszm_ZoneVariance_Apex	0.015
ngtdm_Busyness_Apex	1.381
ngtdm_Coarseness_Apex	-2782.771041
ngtdm_Complexity_Apex	0.000275014
gldm_GrayLevelNonUniformity_Apex	-0.011243202
gldm_LargeDependenceHighGrayLevelEmphasis_Apex	-0.005852267
gldm_LargeDependenceLowGrayLevelEmphasis_Apex	-4.484751303
firstorder_10Percentile_Base	-0.221789269
firstorder_Energy_Base	-2.31411E-08
firstorder_InterquartileRange_Base	-0.191553975
firstorder_Kurtosis_Base	0.28
firstorder_Minimum_Base	-0.10033044
firstorder_Uniformity_Base	511.824
shape_Elongation_Base	9.012
shape_Flatness_Base	-22.76796896
shape_LeastAxisLength_Base	-0.491363729
shape_MajorAxisLength_Base	-0.023559668
shape_Maximum2DDiameterColumn_Base	0.046

shape_Maximum2DDiameterRow_Base	-0.017964975
shape_Maximum2DDiameterSlice_Base	0.095
shape_Maximum3DDiameter_Base	-0.198770216
shape_MinorAxisLength_Base	-0.457096178
shape_Sphericity_Base	-2.336855096
shape_SurfaceArea_Base	0.003600587
shape_SurfaceVolumeRatio_Base	-4.416308449
glcm_ClusterProminence_Base	-3.5651E-06
glcm_Contrast_Base	-0.171013698
glcm_Correlation_Base	0.145
glcm_Imc1_Base	35.684
glcm_Imc2_Base	-7.574759687
glcm_JointAverage_Base	0.861
glcm_JointEnergy_Base	-958.2169646
glcm_MCC_Base	7.3
glcm_MaximumProbability_Base	-734.7529329
glrlm_RunVariance_Base	-154.8836307
glrlm_ShortRunHighGrayLevelEmphasis_Base	-0.021418708
glszm_GrayLevelNonUniformityNormalized_Base	-628.9855369
glszm_LargeAreaHighGrayLevelEmphasis_Base	0.000268432
glszm_LargeAreaLowGrayLevelEmphasis_Base	-2.395007695
glszm_SizeZoneNonUniformity_Base	-0.002338366
glszm_SmallAreaEmphasis_Base	36.437
ngtdm_Busyness_Base	-0.888311909
ngtdm_Complexity_Base	6.33956E-05
ngtdm_Contrast_Base	50.932
ngtdm_Strength_Base	-0.323326191
gldm_DependenceNonUniformityNormalized_Base	-34.53125393
gldm_LargeDependenceLowGrayLevelEmphasis_Base	37.811
gldm_LowGrayLevelEmphasis_Base	371.773

The Radiomics features named as: Feature type_Feature name_Extraction area. WP: whole prostate.
